# Transition Metal-Based Dimeric Metallosurfactants: From Organic–Inorganic Hybrid Structures and Low-Dimensional Magnets to Metallomicelles

**DOI:** 10.1021/acs.inorgchem.4c01550

**Published:** 2024-06-17

**Authors:** Mirta Rubčić, Mirta Herak, Leona Zagorec, Darija Domazet Jurašin

**Affiliations:** † Faculty of Science, Department of Chemistry, University of Zagreb, Horvatovac 102a ,Zagreb HR-10000, Croatia; ‡ Department for Research of Materials Under Extreme Conditions, Institute of Physics, Bijenička cesta 46 ,Zagreb HR-10000, Croatia; § Faculty of Chemical Engineering and Technology, University of Zagreb, Trg Marka Marulića 19 ,Zagreb HR-10000, Croatia; ∥ Division of Physical Chemistry, Ruđer Bošković Institute, Bijenička 54 ,Zagreb HR-10000, Croatia

## Abstract

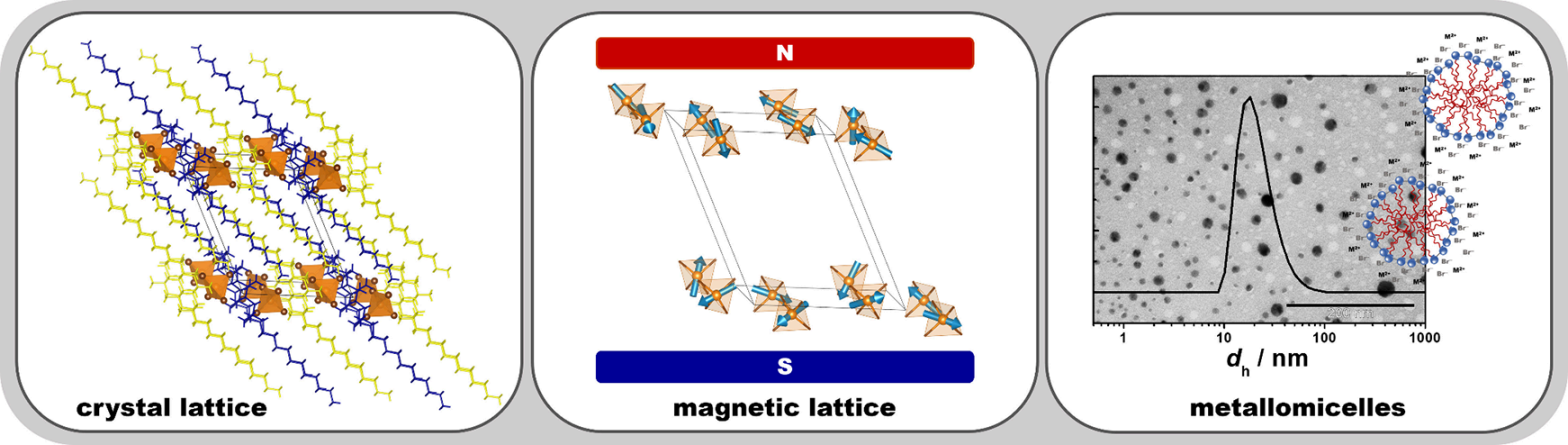

The dimeric (gemini) as well as metallosurfactants exhibit enhanced physicochemical properties compared with conventional surfactants. By uniting the benefits of both, a series of novel dimeric metallosurfactants of the type (12–2–12)[MBr_4_] (M = Co, Ni, Cu and Zn) was successfully prepared by the reaction of the dimeric surfactant bis(*N*,*N*-dimethyl-*N*-dodecyl)ethylene-1,2-diammonium dibromide, 12–2–12, and the MBr_2_ salt. Structures and magnetic properties of the materials were studied comprehensively in the solid state, while their micellization was explored in solution. The obtained results unveil that the incorporation and the choice of transition metal more significantly influence surfactants’ structures ((12–2–12)^2+^ cations adopt V-, U-, or *trans*-conformations) and the magnetic features (metal ions form 1D or 2D magnetic lattice) than their solution properties. However, all synthesized metallosurfactants display improved self-assembly properties compared with the metal-free precursor. The investigated systems represent a fruitful platform for the development of multifunctional materials as they are simple to produce, can be obtained in high yields, and show advanced properties both in solution and in the solid state. Notably, this work unveils a simple approach to the design and synthesis of novel low-dimensional magnetic systems of great potential for future spintronic and optoelectronic devices.

## Abstract 2

The dimeric (gemini) as well as metallosurfactants exhibit enhanced physicochemical properties. By uniting the benefits of both, a series of dimeric metallosurfactants of the type (12-2-12)[MBr_4_] (M = Co, Ni, Cu, Zn) was prepared. Comprehensive studies revealed that the incorporation and choice of metal significantly influence surfactants’ structures and the magnetic features; metal ions form 1D or 2D magnetic lattice, and (12-2-12)^2+^ cations adopt V, U-, or *trans*-conformations. Additionally, all synthesized metallosurfactants display improved self-assembly properties.

## Introduction

Surfactants are organic, amphiphilic molecules that possess unique physicochemical properties both in solution and in the solid state. In solutions, they adsorb at all available interfaces and self-assemble in a variety of aggregates ranging from nano to microscale such as micelles, bilayers, vesicles, and liquid crystalline phases.^1^ Due to the distinctive properties of their aqueous solutions, they are essential in everyday use as well as in numerous industrial and technological processes, such as cleaning, solubilizing, dispersing, emulsifying, wetting, and foaming agents.^1−3^ Moreover, they contributed significantly to many research fields, especially in nanotechnology, owing to their crucial role in stabilizing interfaces and nanostructures.^2,4^

Despite the fact that surfactants are notoriously resistant to the growth of crystals suitable for X-ray analysis, many of them in the solid state yield well-formed crystals.^5^ Due to their intrinsic amphiphilic character, the resulting structures usually appear to be lamellar with alternating head-to-head or tail-to-tail arrangements, i.e., the polar heads and hydrophobic chains are both organized in bilayers but segregated from each other. Numerous bilayers stacked together in one direction form the surfactants’ bulk crystalline phase.^5^ This structural anisotropy and the possibility of two-dimensional confinement of functional ions make surfactants ideal candidates for the design of organic–inorganic hybrid materials.^6,7^ The introduction of metal ions, such as transition metals or lanthanides, represents one of the viable routes toward the development of surfactant-based hybrid structures, which display novel or modulated properties, e.g. magnetic, electrical, and/or optical, as compared to their building blocks.^8−11^ Such hybrids have in particular shown great potential for the development of low-dimensional magnetic structures (e.g., 0D, 1D, or 2D) through the incorporation of magnetic ions. Because of their outstanding properties, two-dimensional (2D) materials have recently become the focus of various materials research areas, with special emphasis on 2D magnetic materials for their potential in future applications in spintronics and optoelectronics.^12−16^

Dimeric, or often called gemini, surfactants are compounds that have proven to be very efficient in various application domains.^3,17,18^ They are usually denoted as *m*-*s*-*m*, where *m* represents the number of carbon atoms in hydrophobic chains and *s* is the number of carbon atoms in the spacer. Unlike conventional single-chain surfactants, they are made up of two amphiphilic moieties covalently linked at the level of the head groups, or very close to them, by a spacer group. Consequently, such compounds exhibit enhanced physicochemical properties compared to their monomeric counterparts.^3,17,18^

Another class of surfactants that has drawn much attention lately is metallosurfactants.^19−21^ This term is commonly used to designate compounds that behave as surfactants and contain a metal in their molecular structures as part of the headgroup (type 1), hydrophobic tail (type 2), or counterion (type 3).^22^ Recent reports have revealed that metallosurfactants exhibit enhanced physicochemical properties compared to conventional surfactants.^19−21^ For this reason, metallosurfactants have been studied for a broad spectrum of applications such as catalysis,^23^ anticancer, and antimicrobial agents,^24,25^ in nanoparticles synthesis,^26−28^ drug-delivery,^29,30^ and templates for mesoporous materials.^31,32^ When considering, in particular, dimeric surfactants, only two types of their metalloderivatives have been reported in the literature. Yi et al. have investigated a homologous series of dimeric surfactants: *m*-2-*m*[XCl_3_Br] (*m* = 14, 16, 18) with X = Fe, Ce, and Gd.^33^ They have shown that both the magnetic counterions and alkyl chain lengths affected surface activities, magnetism, and thermotropic phases of the investigated compounds. The second dimeric metallosurfactant described in the literature with [FeCl_4_]^−^ counterion and hydroxyl group in the spacer has proven to be an effective magnetic deep eutectic catalyst for pyrroles synthesis.^34^

Considering all the factors mentioned above, the aim of this research was to design and explore targeted properties of dimeric metallosurfactants based on transition metals, both in the solid state and in solution. As a dimeric surfactant we chose bis(*N,N*-dimethyl-*N*-dodecyl)ethylene-1,2-diammonium dibromide, 12–2–12, due to its high surface activity, good antibacterial properties and relatively straightforward synthesis and purification.^17,18^ Combining it with the appropriate bromide salt of the selected metal M, we obtained a series of novel metallosurfactants of the type(12–2–12)[MBr_4_] (M = Co, Ni, Cu, and Zn), in solvated and nonsolvated forms, along with the material (12–2–12)_2_[NiBr_2_(H_2_O)_4_]Br_4_·2H_2_O. The obtained results unveil that all investigated metallosurfactants exhibit enhanced physicochemical properties in both solution and in the solid state compared to their metal-free counterpart. To the best of our knowledge, this study is the first to explore to this extent the solid-state properties of dimeric metallosurfactants and quaternary ammonium metallosurfactants in general, via single crystal X-ray diffraction and magnetic measurements. Finally, this work establishes dimeric metallosurfactants as a new class of promising materials with 2D magnetism, where surfactants’ versatility in the formation of different structures opens up a new route toward a true tailoring of magnetic properties in materials.

## Experimental Section

### Materials

*N*,*N*,*N*′,*N*′-Tetramethylethylenediamine (≥99,5%, Sigma-Aldrich) and 1-bromododecane (97%, Sigma-Aldrich) were used for the synthesis of precursor, dimeric surfactant, 12–2–12. Cobalt(II) bromide (≥97%, Thermo Scientific), nickel(II) bromide (≥98%, Sigma-Aldrich), copper(II) bromide (≥99%, Thermo Scientific), and zinc(II) bromide (≥98%, Thermo Scientific) were used for the synthesis of dimeric metallosurfactants, (12–2–12)[MBr_4_]. For synthesis and purification, absolute ethanol (Honeywell Riedel-de Han), methanol (Kemika, Croatia), and acetonitrile (Kemika, Croatia) were used. In addition to the aforementioned solvents, diethyl ether (Kemika, Croatia) was used for single crystals growth. All of the chemicals were used without further purification.

### Synthesis of Dimeric Metallosurfactants (12–2–12)[MBr_4_]

All dimeric metallosurfactants (12–2–12)[MBr_4_] were synthesized via a two-step process. Initially, dimeric surfactant 12–2–12 was prepared as described previously.^35,36^ In short, a reaction mixture of 1-dodecyl bromide and *N*,*N*,*N*′,*N*′-tetramethylethylenediamine in a 2:1 molar ratio in acetonitrile was held at 80 °C for 50 h. After cooling to room temperature, acetonitrile was evaporated using a rotary evaporator, and the compound was isolated as a white solid powder, which was further recrystallized from acetonitrile. All the corresponding metallosurfactants were prepared in high yields from a reaction between respective metal bromide (CoBr_2_, NiBr_2_, CuBr_2_, and ZnBr_2_) and 12–2–12 in 1:1 molar ratio. For each synthesis, approximately 5 g (8.314 mmol) of 12–2–12 and 2 g (∼ 9 mmol) of metal bromide were dissolved and refluxed in 100 mL of absolute ethanol at 70–80 °C for 3 h. Similar protocols have been published in the literature for the synthesis of metallosurfactants from single-chain precursors.^22,37,38^ The solvent was evaporated using a rotary evaporator, and metal complexes were isolated as solid powders with respective colors. Before further analyses, all synthesized compounds were recrystallized from methanol or acetonitrile. The compound characterization (CHN, FTIR, TGA) is given in Supporting Information (SI).

### Methods

#### Single Crystal Analysis

Single crystals of (12–2–12)[MBr_4_], M = Co, Cu, Zn,(12–2–12)[MBr_4_]·MeOH, M = Cu, Co, and (12–2–12)[NiBr_4_]·CH_3_CN, suitable for single-crystal X-ray analysis were grown from nearly saturated methanol or acetonitrile solutions by slow evaporation or a vapor diffusion technique. All solvates were stored in the mother liquor before analysis. The nonsolvates were isolated as sparse crystals after complete solvent evaporation. Interestingly, on prolonged standing of acetonitrile suspensions containing(12–2–12)[NiBr_4_]·CH_3_CN, a stable yellow hydrate of the formula (12–2–12)_2_[NiBr_2_(H_2_O)_4_]Br_4_·2H_2_O was harvested along with previously reported [Ni(H_2_O)_4_(CH_3_CN)_2_]Br_2_.^39^

Diffracted intensities were collected on a Rigaku XtaLAB Synergy diffractometer equipped with a Dualflex source (Cu *K*α radiation, λ = 1.54184 Å) and a HyPix detector using ω-scans. Data were collected at 170 K, except for the (12–2–12)_2_[NiBr_2_(H_2_O)_4_]Br_4_·2H_2_O structure, for which data were gathered at 293 K. The collected data were processed using the CrysAlis program package.^40^ A summary of the general crystallographic data is presented in Table S1. Crystallographic data for the structures are deposited in the Cambridge Structural Database under the CCDC numbers: 2329174–2329180.

#### Magnetic Measurements

The magnetization was measured using the SQUID VSM option of the Quantum Design (QD) Magnetic Properties Measurement System MPMS3. The samples were mounted inside a QD VSM capsule for powder samples, which was placed in a QD brass sample holder. The temperature dependence of magnetization was measured in the applied magnetic field of μ_0_*H* = 0.1 T in the temperature range from 2 to 300 K. The magnetic field dependence of magnetization in the magnetic field range of ±7 T was measured at *T* = 1.8 K or *T* = 2 K.

#### Preparation of Solutions

All of the required solutions were prepared in Milli-Q water using volumetric flasks. All the solutions were thermostated at 25 °C before measurements.

#### Electrical Conductivity and Surface Tension Measurements

The electrical conductivity (κ) measurements were performed with a Conductivity Meter (Methron, Switzerland) in a temperature-controlled double-walled glass container with a circulation of water. The surface tension (γ) measurements were carried out with an Interfacial Tensiometer K100 (Krüss, Germany) using the Du Noüy ring method. These values then were corrected by using the Huh and Mason factors. All measurements were conducted at 25 °C.

#### Light Scattering and Zeta Potential Measurements

The size distribution and zeta potential of (12–2–12)[MBr_4_] micelles were determined by means of dynamic light scattering (DLS) and electrophoretic light scattering (ELS) techniques using a photon correlator spectrophotometer equipped with a 532 nm “green“ laser (Zetasizer Nano ZS, Malvern Instruments, UK). The hydrodynamic diameter (*d*_h_) of micelles was obtained as a value at the peak maximum of the size volume distribution. The zeta potential (ζ) was calculated from the measured electrophoretic mobility by means of the Henry equation using the Smoluchowski approximation. Each sample was measured 6–8 times, and the results were expressed as the average value and standard deviation. The data processing was done by Zetasizer software version 7.13 (Malvern instruments).

#### Transmission Electron Microscopy (TEM)

For TEM analysis, a drop of metallosurfactant solution was placed on the copper grid covered with the hollow Formvar membrane. The excess solution was removed with filter paper. Transmission electron microscopy images were acquired by using a JEOL JEM 1010 transmission electron microscope (JEOL, Tokio, Japan) operated at 80 kV.

A more detailed description of the experimental setups and data interpretation can be found in the Supporting Information (SI).

## Results and Discussion

### Crystal Structures

Altogether, seven novel crystal structures were determined for the studied series of dimeric metallosurfactants (Table S1). Three of these were nonsolvates with the general formula (12–2–12)[MBr_4_] (M = Cu, Co, Zn), while two were methanol solvates of the type (12–2–12)[MBr_4_]·MeOH (M = Cu, Co). For the nickel-based metallosurfactants, structures of acetonitrile solvate, (12–2–12)[MBr_4_]·CH_3_CN, and a hydrate of a complex type of salt, (12–2–12)_2_[NiBr_2_(H_2_O)_4_]Br_4_·2H_2_O, were resolved. Interestingly, except for the (12–2–12)[ZnBr_4_]·MeOH, whose structure could not be established due to inadequate crystal quality, for the Co and Cu analogs solvates were more stable and prevailing forms obtained from the mother liquors. Usually, crystal hydrates are formed when strongly polar surfactants are in question, whose shape does not allow them to pack densely without water molecules present.^5^ Similar reasoning can be applied for the methanol solvates, where solvate molecules interact with polar/ionic functional groups, assuming at the same time a space-filling function and stabilizing the overall crystal packing.

**Figure 1:**
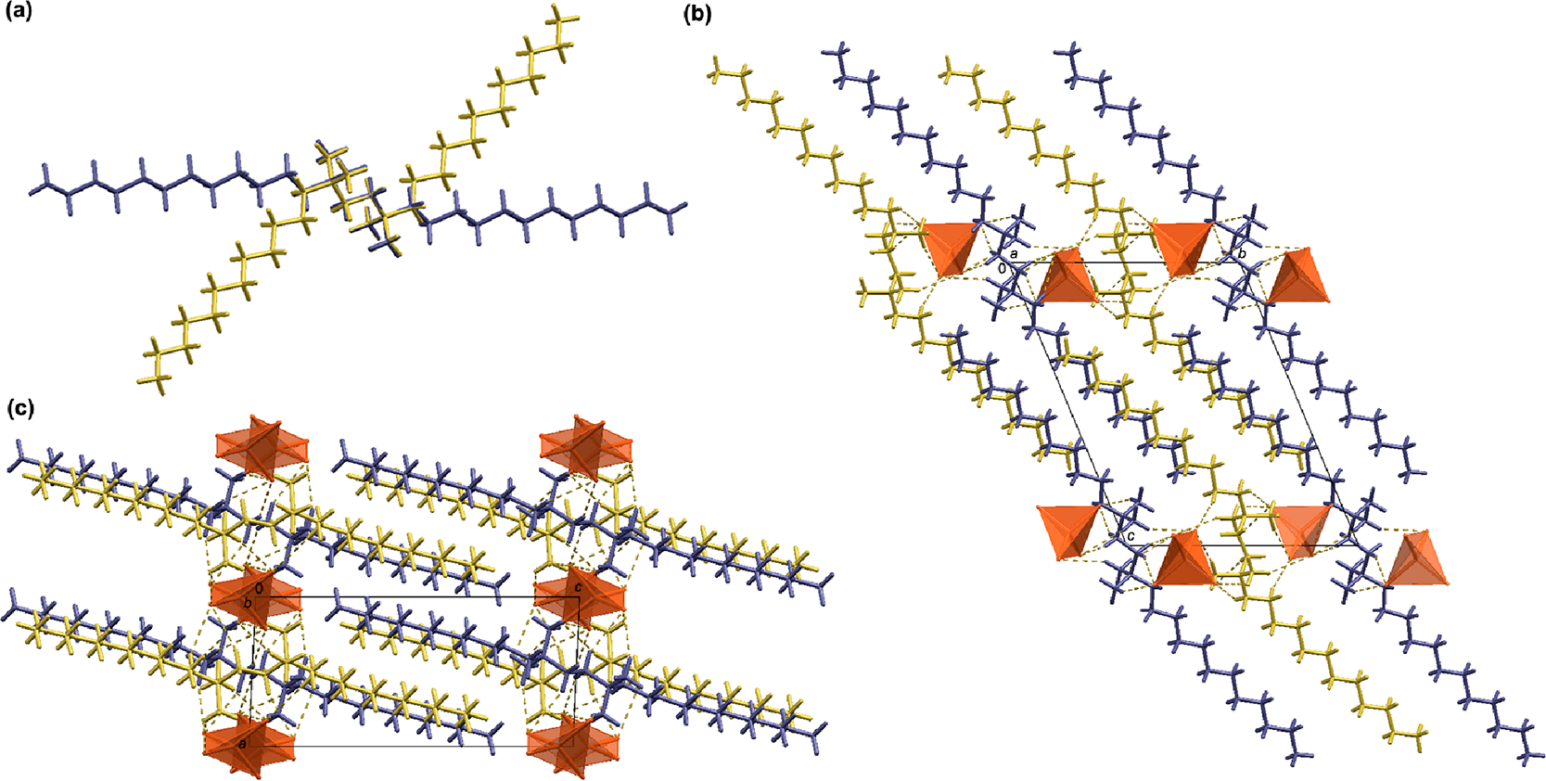
(a) Overlay diagram of the two symmetrically nonequivalent (12–2–12)^2+^ cations (yellow-N1 containing cation and blue-N2 containing cation) in (12–2–12)[CuBr_4_]. The diagram was constructed by overlapping ethyl spacers of the two symmetrically nonequivalent (12–2–12)^2+^ cations. Crystal packing in (12–2–12)[CuBr_4_] is shown down the: (b) *a*-axis and (c) *b*-axis. In (b) and (c), [CuBr_4_]^2–^ anions are presented in a polyhedral style. C–H···Br interactions are highlighted by yellow dashed lines.

When considering nonsolvates of the type (12–2–12)[MBr_4_], it can be seen that the variation in the metal atom within the complex anions does not alter significantly molecular packing, as all members crystallize in the triclinic *P*-1 space group with comparable unit cell dimensions (Table S1). The asymmetric units in all (12–2–12)[MBr_4_] structures contain one tetrabromometallate anion [MBr_4_]^2–^ and halves of the two symmetrically nonequivalent cations (12–2–12)^2+^ cations (Figure S2). Irrespective of the chosen metal, in all cases, a typical lamellar structure with alternating hydrophilic (consisting of the ammonium headgroups with quaternary ammonium cations and inorganic counterions) and hydrophobic regions (composed of dodecyl tails) is formed (Figures 1, S3–4). The dodecyl chains adopt a fully extended, all-*trans* conformation, with the two dodecyl tails forming a 180° torsion angle and aligning themselves in the direction of the longest axis and parallel to one another. In short, the overall structure of (12–2–12)[MBr_4_] in crystals can be described as tilted, interdigitated bilayers, the thickness of which equals the dimension of the unit cell’s *c* axis ∼18 Å. The conformations of the (12–2–12)^2+^ cations observed in the nonsolvate forms are comparable to those of previously reported bromide, iodide, and the triiodide salts based on the same cation.^41,42^

In all (12–2–12)[MBr_4_] structures, the two symmetrically nonequivalent cations (12–2–12)^2+^ assume noticeably different conformations, which can be coupled with the energetically facile rotation around the C–C bond (Figure 1a, Figures S3a and S4a). The most significant differences between (12–2–12)[CoBr_4_] and (12–2–12)[ZnBr_4_] structures on one hand and (12–2–12)[CuBr_4_] on the other are found in the deformation of the [MBr_4_]^2–^ units. While the [CuBr_4_]^2–^ anion exhibits a distorted tetrahedral geometry, which is evident from the relevant τ_4_ value of 0.75 (Table S2), the τ_4_ values for [CoBr_4_]^2–^ and [ZnBr_4_]^2–^ being 0.94 and 0.95, respectively, suggest that these anions adopt nearly ideal tetrahedral geometry (Tables S3 and S4). In the (12–2–12)[MBr_4_] structures, [MBr_4_]^2–^ units are associated with the (12–2–12)^2+^ cations via hydrogen bonds of the C–H···Br type (Table S2).

**Figure 2:**
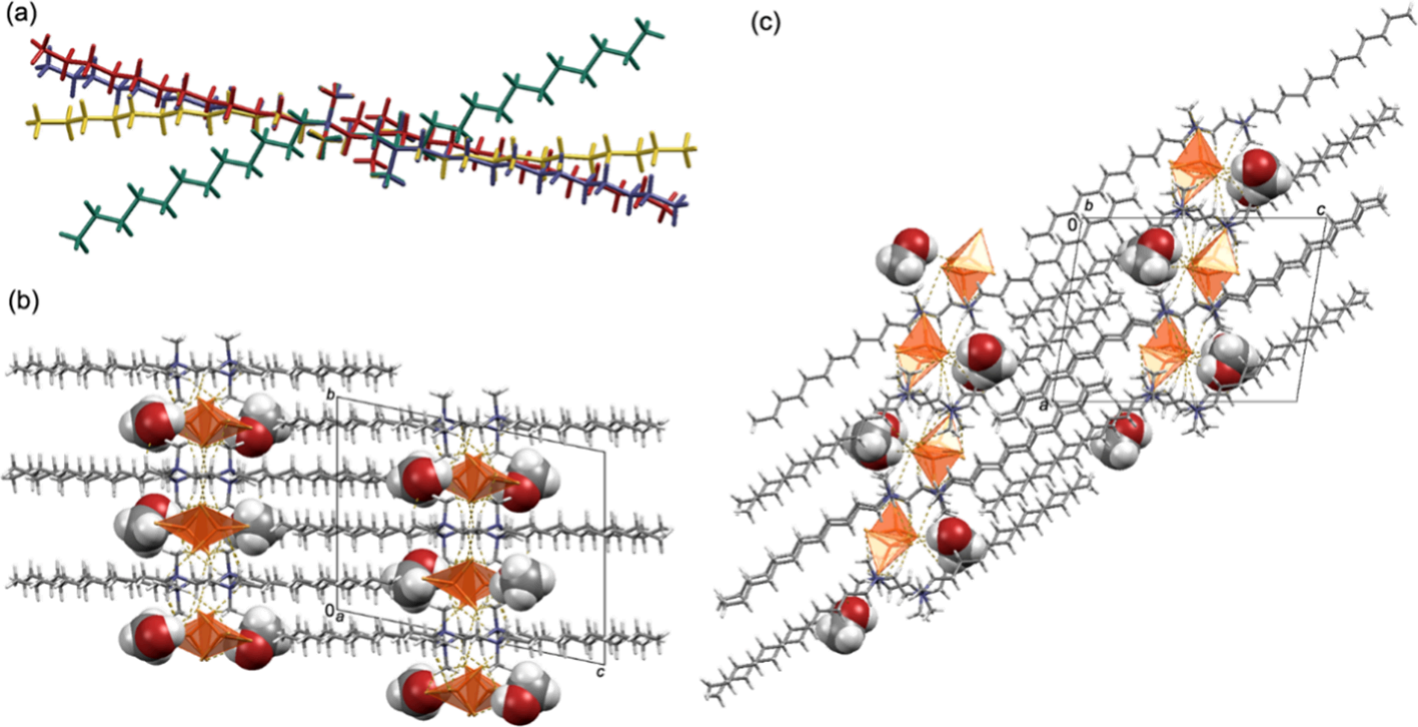
(a) Overlay diagram of the four symmetrically nonequivalent (12–2–12)^2+^ cations (red-N3, yellow-N4, green-N1 and blue-N2) in (12–2–12)[CuBr_4_]·MeOH. The diagram was constructed by overlapping central ethyl spacers of the four symmetrically nonequivalent (12–2–12)^2+^ cations. Crystal packing in (12–2–12)[CuBr_4_]·MeOH is shown down the (b) *a*-axis, and (c) *b*-axis. In (b) and (c), [CuBr_4_]^2–^ anions are presented in the polyhedral style, while the methanol molecules are presented in the spacefill style.

**Figure 3:**
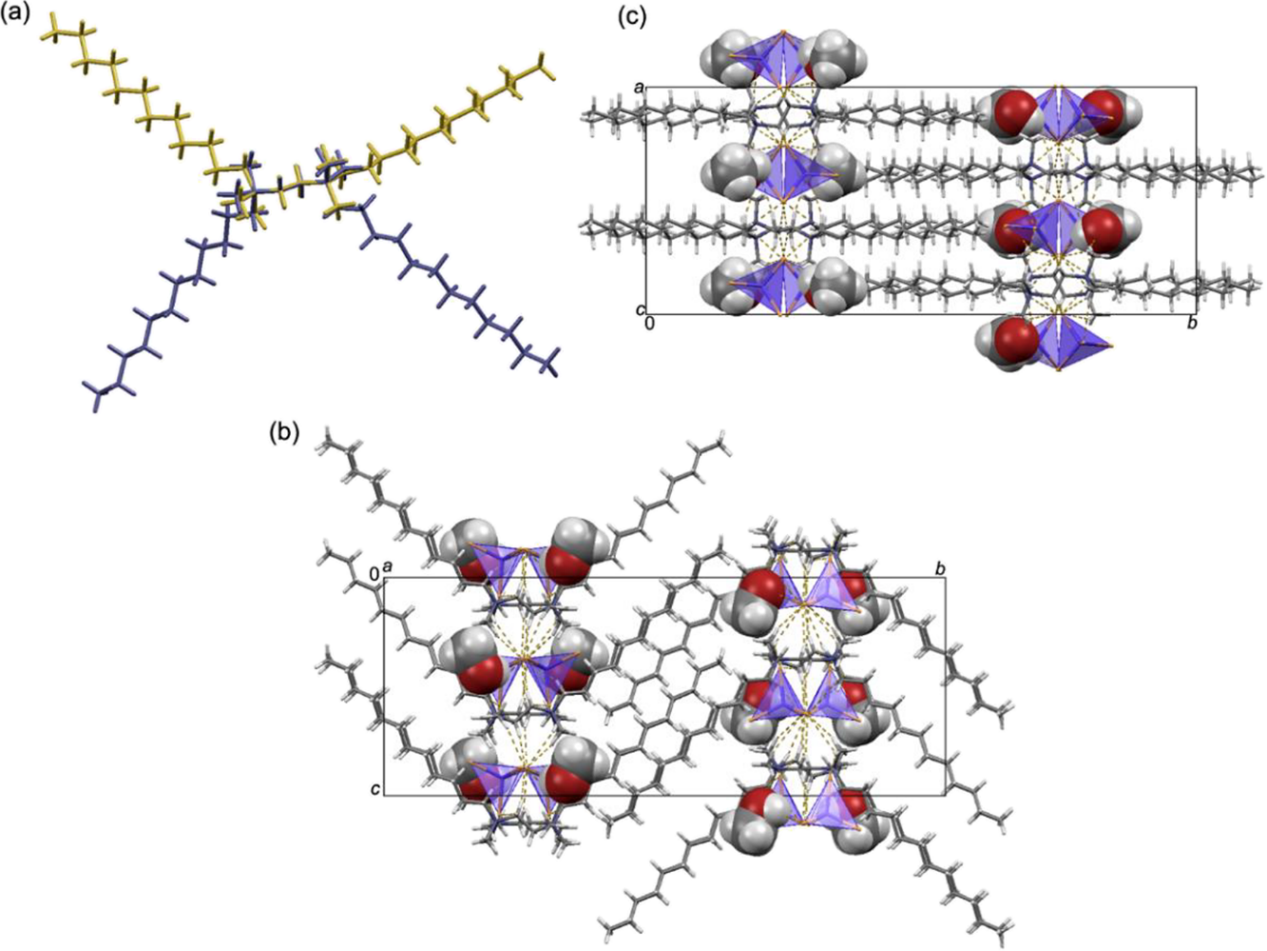
(a) Overlay diagram of the two symmetrically nonequivalent (12–2–12)^2+^ cations (blue – N1,N2 containing cation; yellow – N3,N4 containing cation) in (12–2–12)[CoBr_4_] · MeOH. The diagram was constructed by overlapping central ethyl spacers of the two symmetrically nonequivalent (12–2–12)^2+^ cations. Crystal packing in (12–2–12)[CoBr4]·MeOH shown down the (b) *a*-axis and (c) *c*-axis. In (b) and (c), [CoBr_4_]^2–^ anions are presented in the polyhedral style, while the methanol molecules are presented in the spacefill style. C–H···Br and O–H···Br hydrogen bonds are highlighted by yellow dashed lines.

A greater difference in crystal structures was observed in the solvated forms of the investigated metallosurfactants. In the (12–2–12)[CuBr_4_]·MeOH, which crystallizes in the *P*-1 space group (Table S1), there are four symmetrically nonequivalent (12–2–12)^2+^ cations lying on inversion centers with distinctly different conformations (Figure 2a). As in nonsolvated structures, all dodecyl chains adopt fully extended, all-*trans* conformation with the two dodecyl arms being maximally rotated to each other and forming a 180° torsion angle (Figure 2). In contrast, (12–2–12)^2+^ cations in the (12–2–12)[CoBr_4_]·MeOH adopt a V-shaped form (Figure 3), similar to that observed in the previously reported structure of the bis(*N*,*N*-dimethyl-*N*-dodecyl)ethylene-1,2-diammonium picrate, where the cations assume a V-shaped form with the two dodecyl arms creating a torsion angle of *ca* 60°.^43^ Here, the two symmetrically nonequivalent cations slightly differ in the corresponding torsion angles, one being *ca* 30° and the other *ca* 40°. The asymmetric unit of (12–2–12)[CuBr_4_]·MeOH contains two symmetrically nonequivalent [CuBr_4_]^2–^ anions, which are distorted from ideal tetrahedral geometry to a similar extent (Table S5). On the other hand, in (12–2–12)[CoBr_4_]·MeOH structure [CoBr_4_]^2–^ anions assume a fairly tetrahedral geometry (Figure 3 and Table S5). Due to the presence of methanol molecules in both structures, besides the hydrogen bonds of the C–H···Br type, those of the O–H···Br type are also observed (Tables S5 and S6).

It should be highlighted that although the conformation of (12–2–12)^2+^ cations in (12–2–12)[CoBr_4_]·MeOH differs significantly from those observed in the nonsolvated forms, the thickness of the bilayers remains comparable, equaling approximately one-half of the crystallographic *b*-axis ∼18.4 Å.

An orientation of the hydrophobic chains of the cationic bis-quaternary ammonium surfactants as found in the crystal structure of (12–2–12)[CoBr_4_]·MeOH is very uncommon. Typically, two alkyl chains extend on each side of the spacer plane, as has been observed for other previously discussed structures reported here. Wei et al. have described the first surfactant crystal with a herringbone structure formed from dimeric ammonium molecular ions for the 12–3(OH)–12 compound.^44^ They attributed it to the presence of a pendant hydroxyl group at the spacer, which enhances the hydrogen bonding interactions between the neighboring entities.

**Figure 4:**
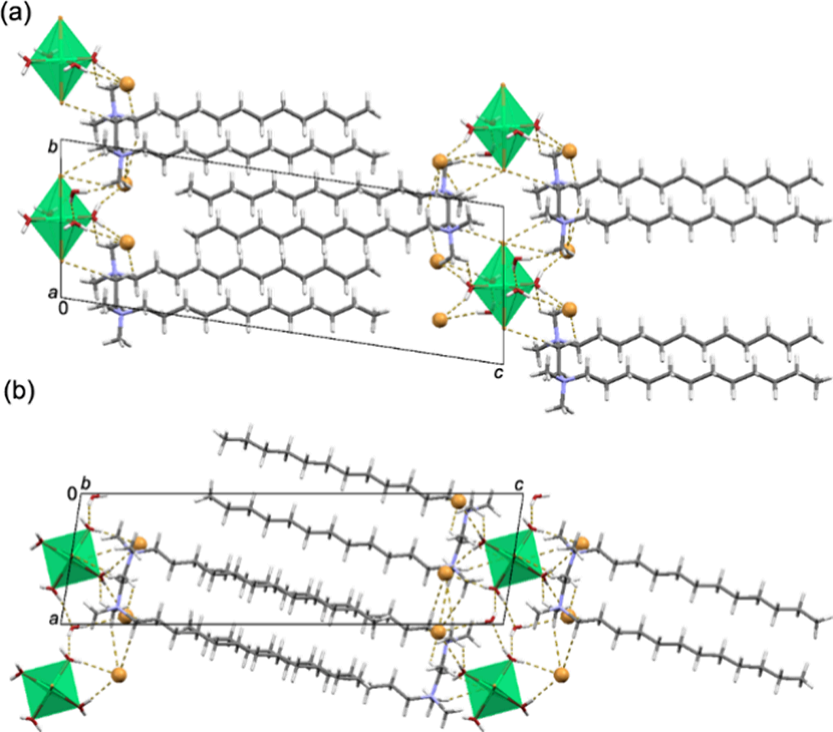
Crystal packing in (12–2–12)_2_[NiBr_2_(H_2_O)_4_]Br_4_·2H_2_O shown down the (a) *a*-axis and (b) *b*-axis. In (a) and (b), neutral [NiBr_2_(H_2_O)_4_] octahedral units are presented in a polyhedral style, while bromide anions are presented as large yellow spheres. C–H···O, C–H···Br, O–H···Br, and O–H···O hydrogen bonds are highlighted by yellow dashed lines.

When considering nickel-based metallosurfactants, it has to be highlighted that from methanol solutions, solvate of the type (12–2–12)[NiBr_4_]·MeOH could not be obtained. On the other hand, when acetonitrile was used as a solvent, (12–2–12)[NiBr_4_]·CH_3_CN was obtained in the form of very thin, air-sensitive, plate-like crystals that crystallized in a triclinic *P*-1 space group (Table S1). The crystal structure of this solvate is to some extent comparable with (12–2–12)[CuBr_4_]·MeOH and includes altogether four symmetrically nonequivalent (12–2–12)^2+^ cations (Figure S5). Here also, the (12–2–12)^2+^ cations adopt fully extended all-*trans* conformation, with noticeable differences between the symmetrically nonequivalent ones. The two symmetrically nonequivalent [NiBr_4_]^2–^ anions adopt a structure close to ideal tetrahedral geometry, as can be seen from the relevant τ_4_ values being 0.92 and 0.93 (Table S7). In the crystal structure, [MBr_4_]^2–^ units, (12–2–12)^2+^ cations, and acetonitrile molecules associate via C–H···Br and C–H···N interactions. Interestingly, when acetonitrile suspensions containing (12–2–12)[NiBr_4_]·CH_3_CN were left standing for a prolonged time period, a stable yellow hydrate of the formula (12–2–12)_2_[NiBr_2_(H_2_O)_4_]Br_4_·2H_2_O was obtained, along with the previously identified [Ni(H_2_O)_4_(CH_3_CN)_2_]Br_2_.^39^ The crystal structure of (12–2–12)_2_[NiBr_2_(H_2_O)_4_]Br_4_·2H_2_O substantially differs from all other investigated compounds as it contains a neutral [NiBr_2_(H_2_O)_4_] coordination entity, with the Ni atom in octahedral geometry, cations in the U-shaped form with the two dodecyl arms shaping almost 0° torsion angle, and bromides as counterions (Figure 4, Figure S2). To the best of our knowledge, this is the first time that this kind of conformation has been observed for (12–2–12)^2+^ cations. A similar U-shaped conformation of alkyl chains is previously reported for series of *N,N’*-dialkylbenzimidazolium salts with [CuCl_4_]^2–^ anions.^45^ Due to the presence of a number of good hydrogen-bond donors and acceptors in the (12–2–12)_2_[NiBr_2_(H_2_O)_4_]Br_4_·2H_2_O structure, a rich network of C–H···Br, C–H···O, O–H···Br, and O–H···O hydrogen bonds is established (Table S8).

### Magnetic Properties

The magnetic susceptibility of the metal-free precursor 12–2–12 is weakly diamagnetic and temperature-independent with the magnitude on the order of 10^–4^ emu/mol, as expected for a complex with no paramagnetic ions present. The same is true for (12–2–12)[ZnBr_4_], which has a nonmagnetic Zn^2+^ transition metal ion (TMI) with no unpaired electron spins (not shown).

**Figure 5:**
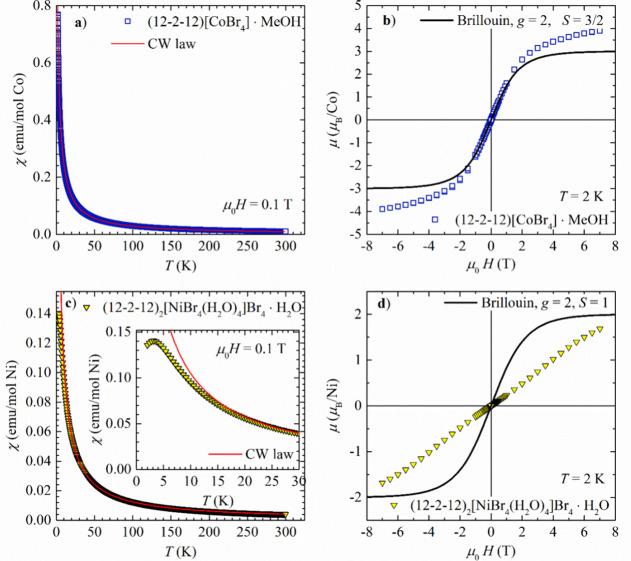
Temperature dependence of magnetic susceptibility of dimeric metallosurfactants: (a) (12–2–12)[CoBr_4_]*·*MeOH and (c) (12–2–12)[NiBr_4_(H_2_O)_4_]Br_4_*·*H_2_O (symbols). Inset in part (c) shows the maximum in low-temperature susceptibility of (12–2–12)[NiBr_4_(H_2_O)_4_]Br_4_*·*H_2_O. The solid red line represents the curve obtained from the fit of susceptibility to the CW law. (b) Magnetic field dependence of magnetic moment μ expressed in μ_B_/Co of dimeric metallosurfactant (12–2–12)[CoBr_4_]*·*MeOH (symbols). (d) Magnetic field dependence of magnetic moment μ expressed in μ_B_/Ni of the dimeric metallosurfactant (12–2–12)_2_[NiBr_2_(H_2_O)_4_]Br_4_·2H_2_O (symbols). The solid black line represents the Brillouin function.

**Figure 6:**
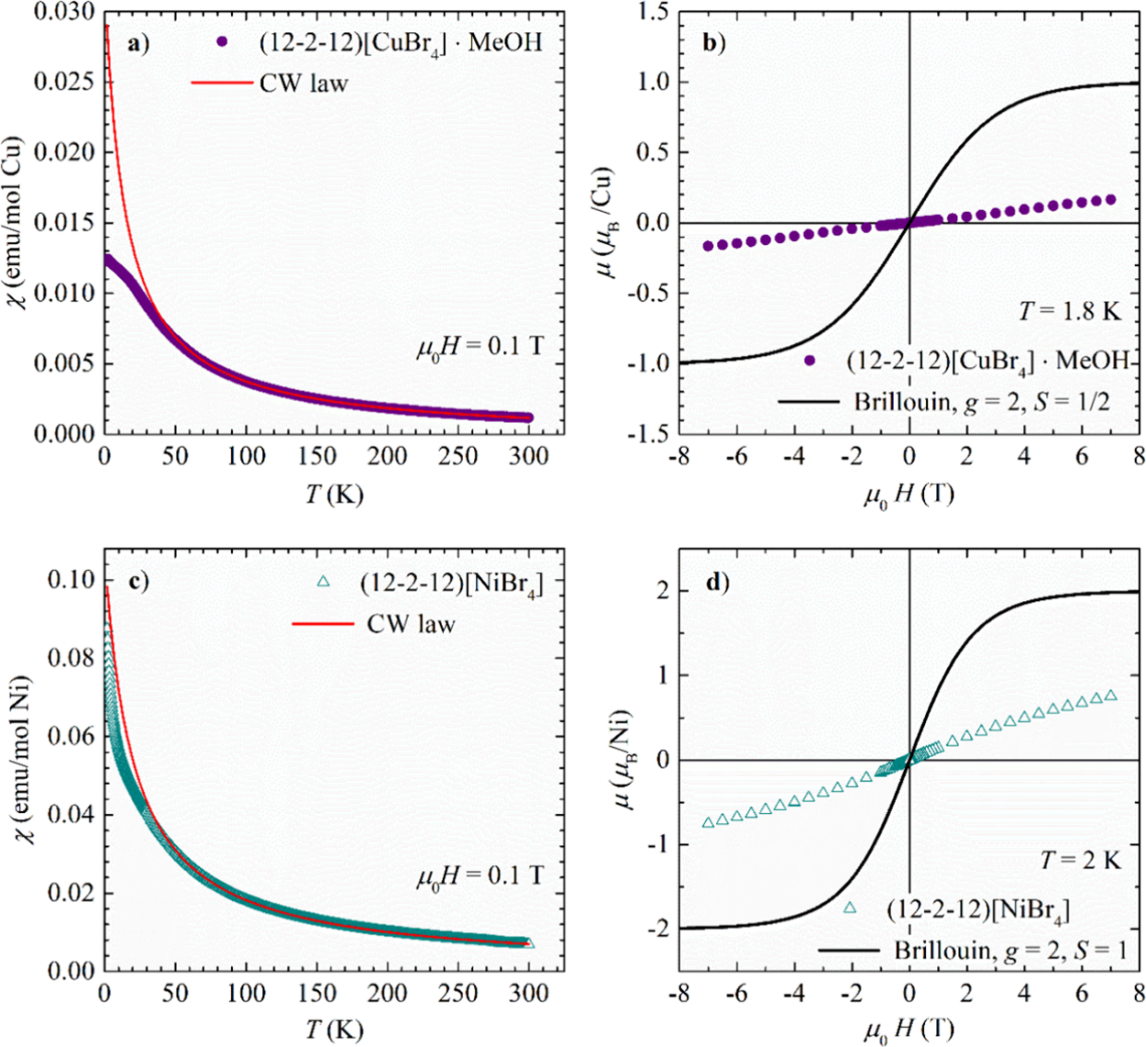
Temperature dependence of magnetic susceptibility of dimeric metallosurfactants: (a) (12–2–12)[CuBr_4_]*·*MeOH and (c) (12–2–12)[NiBr_4_] (symbols). The solid red line represents the curve obtained from the fit of susceptibility to the CW law. (b) Magnetic field dependence of magnetic moment μ expressed in μ_B_/Cu of dimeric (12–2–12)[CuBr_4_]·MeOH measured (symbols). (d) Magnetic field dependence of magnetic moment μ expressed in μ_B_/Ni of dimeric(12–2–12)[NiBr_4_] (symbols). The solid black line represents the Brillouin function.

The temperature dependence of the magnetic susceptibility χ(*T*) and the magnetic field dependence of magnetic moments μ for complexes with magnetic TMI (Cu^2+^, Ni^2+^, and Co^2+^) are shown in Figures 5 and 6. For all compounds, the temperature dependence of magnetic susceptibility χ(*T*) can be well described by the Curie–Weiss law (CW law, see eq S2),^46^ with the inclusion of temperature-independent contribution of susceptibility (see eq S1), in the wide temperature range (50 K ≲ *T* ≤ 300 K) (Figures 5a,c and 6a,c). From the fit of susceptibility to the CW law, we obtain the effective magnetic moment μ_eff_ of the TMI as well as the Curie–Weiss temperature Θ_CW_ (Table 1).

**Table 1:** Parameters Obtained from the Fit of the Susceptibility to the Curie–Weiss Law for Investigated Dimeric Metallosurfactants (12-2-12)[MBr_4_] with Corresponding Dimensionality of Magnetic Lattice, μ_eff_ = Effective Magnetic Moment, and Θ_CW_ = Curie–Weiss Temperature

compound^a^	spin	μ_eff_/μ_B_	Θ_CW_ /K	mag. lattice
(12–2–12)[CoBr_4_] **·** MeOH	3/2	4.848(1)	–1.96(3)	0D
(12–2–12)_2_[NiBr_2_(H_2_O)_4_]Br_4_·2H_2_O	1	3.2037(2)	–2.23(1)	1D
(12–2–12)[CuBr_4_] **·** MeOH	1/2	1.925(1)	–14.0(1)	2D
(12–2–12)[NiBr_4_]	1	4.1522(2)	–20.17(1)	2D

a When interpreting the results of susceptibility measurements, we chose to consider (12–2–12)[CuBr_4_] and (12–2–12)[CoBr_4_] as solvates, as opposed to (12–2–12)[NiBr_4_], which we treated as nonsolvate. Namely, (12–2–12)[CuBr_4_]·MeOH and (12–2–12)[CoBr_4_]·MeOH solvates proved to be fairly stable, although some solvent loss could be expected upon prolonged standing. On the other hand, crystals of (12–2–12)[NiBr_4_]·CH_3_CN, as mentioned in the text, were extremely unstable due to a rapid solvent loss.

The sign of Θ_CW_ determines whether the mean interaction between the magnetic moments is antiferromagnetic (Θ_CW_ < 0) or ferromagnetic (Θ_CW_ > 0), and the magnitude gives the strength of effective interaction expressed in Kelvin.^46^

#### Paramagnetic (12–2–12)[CoBr]_4_·MeOH

The temperature dependence of the magnetic susceptibility χ(*T*) of dimeric metallosurfactant (12–2–12)[CoBr_4_]·MeOH is shown in Figure 5a. From the fit of susceptibility to the CW law, we obtain the effective magnetic moment of μ_eff_ = 4.848(1) μ_B_/Co (Table 1), typical for Co^2+^ ion with spin *S* = 3/2 in a tetrahedral ligand field with incomplete quenching of orbital angular momentum and intermediate spin–orbit coupling.^46^ The obtained Curie–Weiss temperature Θ_CW_ = −1.96(3) K signifies very weak antiferromagnetic interaction between the Co spins.^46^ This is corroborated by the magnetic-field dependence of the magnetic moment measured at *T* = 2 K (Figure 5b). The deviation from the Brillouin function for *S* = 3/2 (solid black line in Figure 5b) is a result of incomplete quenching of orbital angular momentum (magnitude slightly larger than expected for *S* = 3/2) and very weak interaction between the magnetic moments (lack of saturation at the highest applied field). Therefore, (12–2–12)[CoBr_4_]·MeOH can be described as a paramagnetic system forming a magnetic lattice of almost isolated Co^2+^ magnetic moments.

The [CoBr_4_]^2–^· tetrahedra form 2D layers in the *ac* plane of (12–2–12)[CoBr_4_]·MeOH (Figure S6), which are well separated by the (12–2–12)^2+^ cations, which introduce a space with no magnetic moments and effectively allow a formation of a two-dimensional (2D) magnetic lattice. However, the dimensionality of the magnetic lattice is defined by the interactions between the magnetic moments, which are usually superexchange or super-superexchange interactions between the spins of unpaired electrons from the metal (M) via the orbitals of the ligand bridges (Br). In all the studied metallosurfactants, the metal ions do not share ligands of their coordination polyhedra, so only super-superexchange of the type M–Br-Br-M can be realized. The magnitude as well as the sign of super-superexchange interactions between the magnetic moments will be determined by the orientations of the neighboring [MBr_4_]^2–^ tetrahedra as well as Br–Br distances. In (12–2–12)[CoBr_4_]·MeOH, the three unpaired spins *S* = 1/2 of [CoBr_4_]^2–^ are in *t*_2g_ orbitals. Since the system is almost fully paramagnetic, we can conclude that the arrangement of almost perfect [CoBr_4_]^2–^ tetrahedra within the layer (Figure S6) is not favorable for inducing measurable interactions between the spins.

#### 1D Antiferromagnetic (12–2–12)_2_[NiBr_2_(H_2_O)_4_]Br_4_·2H_2_O

The temperature dependence of the magnetic susceptibility of the dimeric metallosurfactant (12–2–12)_2_[NiBr_2_(H_2_O)_4_]Br_4_·2H_2_O is shown in Figure 5c. It can be described by the CW law in the wide temperature range from approximately 15 to 300 K. The fit of the data to CW law gives the effective moment of μ_eff_ = 3.2037 (2) μ_B_/Ni (Table 1), typical for Ni^2+^ in an octahedral environment.^46^ The CW temperature Θ_CW_ = −2.23 K signifies a weak antiferromagnetic interaction between the magnetic moments. However, below *T* ≈ 15 K, the susceptibility decreases more rapidly with decreasing temperature than expected from the CW law and at *T* ≈ 3 K displays a maximum, a clear signature of the low-dimensional antiferromagnetic interactions (inset of Figure 5c). Its presence is further corroborated by the field dependence of magnetization measured at *T* = 2 K (Figure 5d). The measured magnetic moment is almost linear in the magnetic field, contrary to what is expected for free spins *S* = 1 (Brillouin function shown as solid black line in Figure 5d). The moment does not saturate in the highest applied field of 7 T, supporting the presence of weak antiferromagnetic interactions.

**Figure 7:**
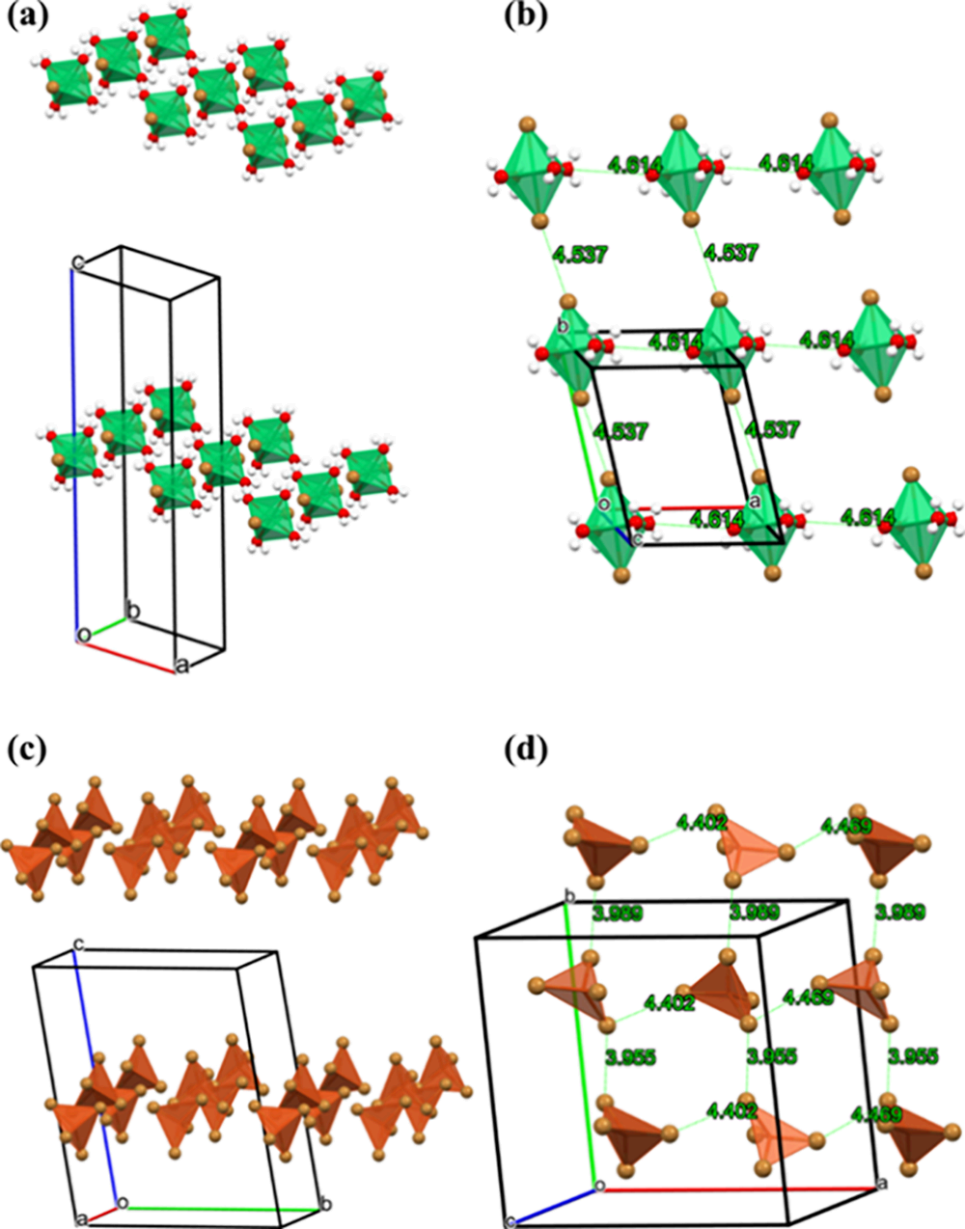
(a) Effective 1D magnetic lattice is established in 2D layer in (12–2–12)_2_[NiBr_2_(H_2_O)_4_]Br_4_·2H_2_O through Ni–Br–Br-Ni super-superexchange interaction. Green octahedra represent NiBr_2_(H_2_O)_4_. (b) Shortest Br–Br and O–O distances in the 2D layer. (c) 2D magnetic lattice in (12–2–12)[CuBr4]*·*MeOH. Orange tetrahedra represent [CuBr_4_]^2–^ anions. The super-superexchange interaction is expected to run along Cu–Br–Br-Cu paths. (d) Shortest Br–Br distances within the layer confined to the *ab* plane. Organic (12–2–12)^2+^ cations are not shown for the sake of clarity.

The NiBr_2_(H_2_O)_4_ octahedra form 2D layers in the *ab* plane, which are well separated by the (12–2–12)^2+^ cations (Figure 7a,b). The Br–Br paths between the NiBr_2_(H_2_O)_4_ octahedra (*d*_Br–Br_ = 4.537 Å) along the *a*-axis enable the unpaired spins from *e*_g_ orbitals of Ni^2+^ to interact via the bromines’ 4*p* orbitals. If super-superexchange was to take place along the *b*-axis, it would be mediated through O–O paths (*d*_O–O_ = 4.614 Å). These distances are too large to give significant overlap of the oxygen 2*p* orbitals, while for bromines’ 4*p* orbitals, overlap should result in significant super-superexchange. Therefore, (12–2–12)_2_[NiBr_2_(H_2_O)_4_]Br_4_·2H_2_O is most likely a realization of a spin *S* = 1 1D Heisenberg antiferromagnet. The observed maximum in susceptibility (inset of Figure 5c) supports this conclusion.^47^

#### 2D Antiferromagnetic (12–2–12)[CuBr_4_] · MeOH and (12–2–12)[NiBr_4_]

The temperature dependence of the magnetic susceptibility of the dimeric metallosurfactant (12–2–12)[CuBr_4_]·MeOH is shown in Figure 6a. It can be described by the CW law in the wide temperature range from approximately 50 to 300 K. The fit of the data to the CW law gives the effective moment of μ_eff_ = 1.925(1) μ_B_/Cu (Table 1), typical for Cu^2+^ ions with spin *S* = 1/2 with quenched orbital angular momentum.^46^ The CW temperature Θ_CW_ = −14 K signifies a moderate antiferromagnetic interaction between the magnetic moments. Below approximately 45 K, the susceptibility increases less rapidly than expected from the CW law. This is evidence of the strengthening of low-dimensional antiferromagnetic interactions, which is further corroborated by the field dependence of magnetic moment measured at *T* = 1.8 K (Figure 6b). The measured moment is significantly suppressed with respect to the value expected for free spins of *S* = 1/2 (Brillouin function plotted by the black solid line in Figure 6b).

The magnetic lattice in (12–2–12)[CuBr_4_]·MeOH is formed by the deformed [CuBr_4_]^2–^ tetrahedra, which are confined in 2D layers in the *ab* plane separated by (12–2–12)^2+^ ions (Figure 7c). The shortest Br–Br distance in Cu–Br–Br-Cu super-superexchange path is 3.955 Å and the next is 3.989 Å along the *b* axis, while along the *a* axis the next shortest distance is 4.402 Å (Figure 7d). This is comparable to 2D antiferromagnetic system bis(2,3-dimethylpyridinium)-tetrabromocuprate (DIMPY),^48^ where the shortest Br–Br distance of 3.905 Å leads to antiferromagnetic superexchange energy of approximately *J*/*k*_B_ = −14 K, and the next shortest Br–Br distance of 4.328 Å results in the superexchange of approximately −10 K.^49^ These values are comparable to the value of Θ_CW_ = −14 K obtained from the fit to the CW law for the average interaction between the spins for (12–2–12)[CuBr_4_]. DIMPY represents a realization of a special 2D magnetic system called spin ladder, one of the several types of low-dimensional magnetic lattices, which host exotic quantum states of matter.^49−52^

The temperature dependence of the magnetic susceptibility of the dimeric metallosurfactant (12–2–12)[NiBr_4_] is shown in Figure 6c. Similar to (12–2–12)[CuBr_4_]·MeOH, the susceptibility obeys the CW law in the temperature range from approximately 50 to 300 K with the effective magnetic moment of μ_eff_ = 4.1522(2) μ_B_/Ni (Table 1), slightly larger than typical Ni^2+^ spin *S* = 1/2 in tetrahedral ligand field, signifying incomplete quenching of the orbital angular momentum.^46^ The CW temperature Θ_CW_ = −20.2 K reveals moderate antiferromagnetic interactions between the spins. The more rapid decrease of susceptibility below approximately 40 K points to the strengthening of low-dimensional antiferromagnetic interactions. This is corroborated by the magnetic-field dependence of magnetic moment measured at *T* = 2 K (Figure 6d), which has a much smaller magnitude than what is expected for noninteracting spins *S* = 1 (Brillouin function plotted by solid black line in Figure 6d). The magnetic lattice in (12–2–12)[NiBr_4_] is formed by [NiBr_4_]^2–^ tetrahedra, which are confined in 2D layers in the *ab* plane separated by (12–2–12)^2+^ ions (Figure S7), very similar to the one in (12–2–12)_2_[CuBr_4_]·MeOH (Figures 7c,d). Therefore, it is not surprising that the magnetic properties of these two complexes are similar as well.

In Table 1, we summarize the results obtained from fitting the data to the CW law for all of the investigated dimeric metallosurfactants. We note that an antiferromagnetic interaction between the magnetic moments of TMI is present in all investigated complexes. This is further corroborated by plotting the χ_spin_ (*T*)·*T* as a function of temperature *T* (Figure S8), where χ_spin_(*T*) represents the spin-only contribution to the susceptibility. In the case of pure paramagnetic response with no interactions, χ_spin_ (*T*)·*T* would be a constant (eq S2). In Figure S8, it is visible that χ_spin_ (*T*)·*T* remains constant at high temperatures for all complexes. However, as the temperature decreases, χ_spin_ (*T*)·*T* also decreases and χ_spin_(*T*) starts to deviate from the CW law, signifying a strengthening of antiferromagnetic interactions. This is typical for low-dimensional antiferromagnetic spin systems in which the antiferromagnetic superexchange interaction between the spins is confined to one dimension (spin chains) or two dimensions (spin layers).^50^ We note that none of the compounds exhibit a phase transition to a long-range ordered magnetic state, despite the observable moderate interactions between the magnetic moments. This is also in line with the expected behavior for low-dimensional magnetic systems.^47,50^

In all the investigated compounds, the (12–2–12)^2+^ cations serve to effectively confine the magnetic metal ions to well-separated 2D planes with virtually no interaction between the spins from different planes. Therefore, the magnetic lattices in these compounds have dimension 2 or less. Here, we point out that the interplay between the conformation of the (12–2–12)^2+^ cations and the ligand environment of a specific metal ion plays a role in determining the dimensionality of the magnetic lattice for the studied metallosurfactants. The V-shaped conformation of organic cations is found in (12–2–12)[CoBr_4_]·MeOH (Figure 3), which has almost purely paramagnetic behavior, suggesting that the orbital overlap of 4*p* orbitals of Br from the neighboring [CoBr_4_]^2–^ tetrahedra is not favorable for mediating super-superexchange between the unpaired spins from *t*_2g_ orbitals of Co^2+^. In (12–2–12)_2_[NiBr_2_(H_2_O)_4_]Br_4_·2H_2_O (Figure 4), where (12–2–12)^2+^ cations adopt a U-shaped conformation, the NiBr_2_(H_2_O)_4_ octahedra orient in such a way that the overlap of the bromines’ 4*p* orbitals of neighboring octahedra forms a 1D magnetic path for interaction of unpaired spins from the *e*_g_ orbitals of Ni^2+^. Finally, in (12–2–12)[CuBr_4_]·MeOH (Figure 2) and (12–2–12)[NiBr_4_] (Figure S5), a similar arrangement of 12–2–12 cations in *trans* conformations and similarity of [CuBr_4_]^2–^ and [NiBr_4_]^2–^ tetrahedra likely results in the similar magnetic Br–Br interaction paths between the unpaired spins from the *t*_2g_ orbitals, which would explain the comparable magnetic properties observed for these two compounds.

We note that all investigated compounds have an underlying 2D magnetic lattice well separated by the surfactants’ cations and therefore hold great potential for the formation of true 2D magnets and antiferromagnets. Finding a way to increase the interactions between the magnetic ions by using structurally different surfactants or, e.g., chemical or physical pressure is the next step forward. The promising applications of 2D magnetic materials are well recognized and include emerging spintronic and spin-dependent optoelectronic devices with increased read/write speed and density.^15^ Finding a way to break the inversion symmetry could lead to ferroelectric as well as magnetoelectric properties and voltage-controlled magnetoelectronics,^16^ of great potential for application in future energy-efficient spintronic devices.

### Self-Assembly Properties

**Figure 8:**
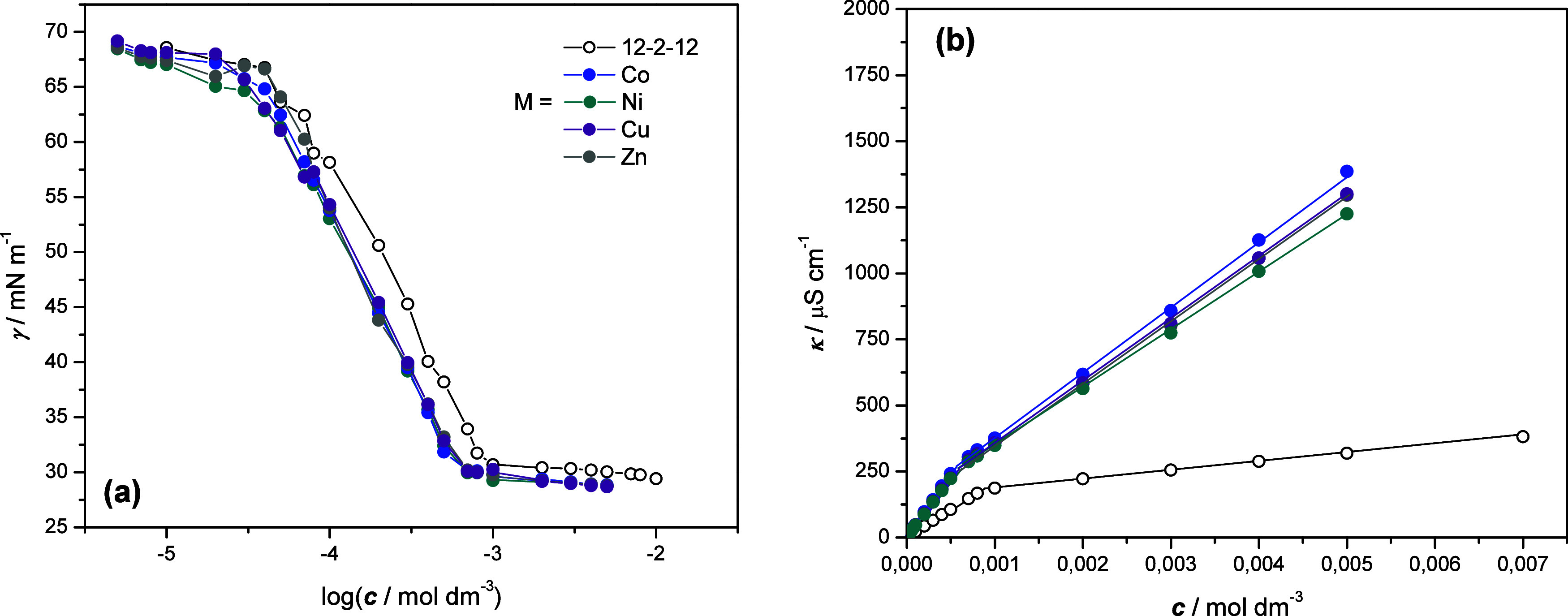
(a) Variation of the surface tension (γ) and (b) electrical conductivity (κ) with the concentration of dimeric metallosurfactants (12–2–12)[MBr_4_] and metal-free precursor 12–2–12. The legend for transition metals is indicated.

Figure 8a illustrates the surface tension (γ) isotherms for a synthesized series of dimeric metallosurfactants, (12–2–12)[MBr_4_], as well as for the dimeric precursor, 12–2–12. The corresponding dependencies of the electrical conductivity (κ) are shown in Figure 8b. All γ vs log *c* curves exhibit a typical pattern with an abrupt change in slope at a concentration corresponding to the cmc. Above the cmc, an almost constant value is observed due to saturation of the air/solution interface. The reported values for dimeric precursor 12–2–12 listed in Table 2 are consistent with the literature.^18,35^

**Table 2:** Critical Micellization Concentrations Obtained from Surface Tension (cmc_γ_) and Conductivity Measurements (cmc_κ_) for (12-2-12)[MBr_4_] Metallosurfactants and Metal-free Precursor 12-2-12 at 25 °C

**compound**	**cmc**_**γ**_**/10**^**–4**^**mol dm**^**–3**^	**cmc**_**κ**_**/10**^**–4**^**mol dm**^**–3**^
12–2–12	8.5	8.9
(12–2–12)[CoBr_4_]	5.7	5.4
(12–2–12)[NiBr_4_]	6.2	5.7
(12–2–12)[CuBr_4_]	6.1	5.7
(12–2–12)[ZnBr_4_]	6.0	5.4

The cmc values obtained from the surface tension (cmc_γ_) and the electrical conductivity (cmc_κ_) measurements were in good agreement for all investigated surfactants (Table 2). As can be seen, the determined cmc values for all dimeric metallosurfactants were lower than those of the metal-free precursor. An equal trend has been reported in the literature for single-chain^53,54^ and double-chain^38,55^ type 3 quaternary ammonium metallosurfactants. However, in the case of the latter, lower cmc values are a result of an increased chain number rather than the introduction of a complex counterion considering that they are synthesized from a single-chain precursor. The differences in the obtained cmc values within the newly synthesized series were not very pronounced and are more or less independent of the metal ion.

**Figure 9:**
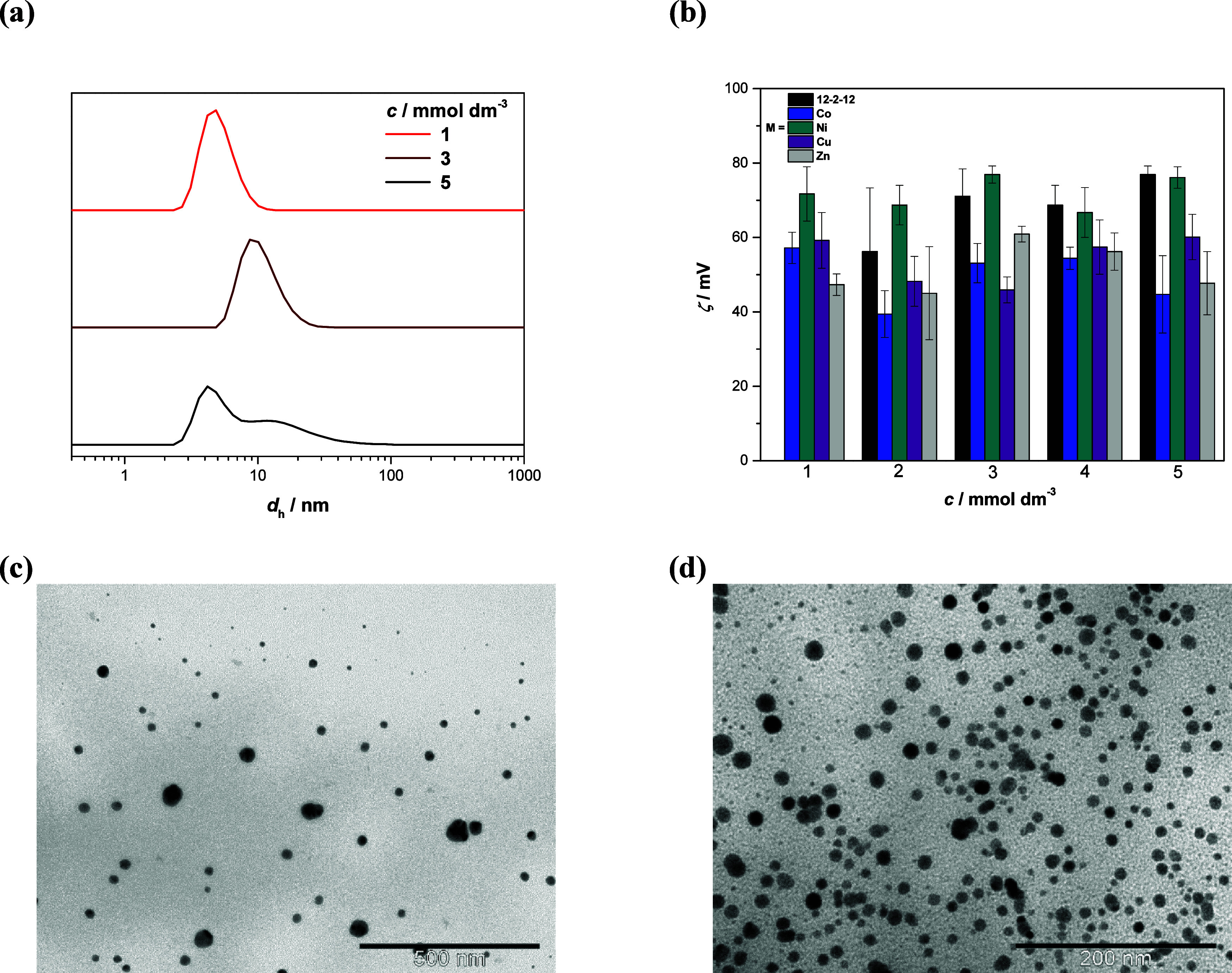
Variation of (a) representative volume size distributions, (b) average zeta potential (ζ) of micelles with the concentration (*c*) for (12–2–12)[MBr_4_] metallosurfactants and metal-free precursor 12–2–12 at 25 °C. Representative TEM images of metallomicelles: (c) *c*((12–2–12)[CuBr_4_]) = 2 × 10^–3^ mol dm^–3^ and (d) *c*((12–2–12)[CoBr_4_]) = 4 × 10^–3^ mol dm^–3^.

It was found that in all (12–2–12)[MBr_4_] systems, the measured average hydrodynamic diameter (*d*_h_) of metallomicelles increases from 4.5 to ∼ 16.0 – 18.0 nm as the concentration increases (Table S9). Furthermore, at the highest measured surfactants concentration (5 × 10^–3^ mol dm^–3^), a bimodal size distribution was observed, with a dominant population having an average *d*_h_ ∼ 6.0 nm, and a secondary population of larger particles with an average *d*_h_ ∼ 20.0 nm (Figure 9a). For these measurements, the volume percentage (vol %) of the measured size populations showed significant fluctuations and a relatively large standard deviation (Table S9). Most likely as a consequence of DLS inherent problem in measuring the size of nonspherical particles. Furthermore, all metallosurfactants’ micelles above 2 × 10^–3^ mol dm^–3^, except in the case of (12–2–12)[NiBr_4_], exhibit less positive zeta potential (ζ) values compared to the 12–2–12 systems (Figure 9b).

One of the most significant properties of dimeric surfactants with short spacers is their tendency to form elongated micelles at relatively low concentrations even without the addition of electrolytes.^17,18^ The differences in aggregation behavior between monomeric and dimeric surfactants are a consequence of bimodal distribution of ionic headgroup distances at the micelle/solution interface, i.e., one distribution maximum corresponds to the thermodynamic equilibrium distance, and the other one corresponds to the length of the spacer.^17^ Therefore, due to the anisotropic shapes of their molecules, dimeric surfactants show a high tendency to form elongated aggregates at very low concentrations. It is not possible to determine the micellar shape by DLS measurements, but based on the obtained *d*_h_ values and available literature data, it can be concluded that a transition occurs at the highest investigated concentration from spherical to cylindrical metallomicelles. In contrast, the micelles in the 12–2–12 system exhibited a monomodal size distribution over the entire concentration range. The chosen representative TEM images for spherical metallomicelles in investigated systems are shown in Figure 9c,d. Dark spots on the micelles’ surface, which originate from metal ions, are visible in all micrographs.

In solution, the [MBr_4_]^2–^ counterions dissociate into M^2+^ (aq) and Br^–^ ions as evident through the observed color change immediately after the complexes dissolve. The pale pink, green, and blue color of the solutions indicate the presence of the corresponding aqua species Co^2+^, Ni^2+^, and Cu^2+^, respectively (Figure S9). In addition, much higher electrical conductivity of the metallosurfactants compared to the dimeric precursor 12–2–12 (Figure 8b) affirms the dissociation of [MBr_4_]^2–^ anions. Heightened ionic strength results in a more effective screening of the repulsive interactions between the quaternary ammonium head groups, thereby leading to a lower cmc and an increase in micelle size. Consequently, the systems behave as if the electrolyte had been added. A similar conclusion was reported by Kaur et al. when studying the organization of metal ions in DTA[CuBrCl_2_] at the air/solution and micelle/solution interfaces.^56^

Understanding the impact of metal ions on micelles’ size and zeta potential is crucial for optimizing their performance in various applications. The ζ potential is an important indicator of stability for colloidal systems.^57^ Although the investigated metallomicelles exhibit somewhat lower ζ potential compared to metal-free systems, their values are still high enough to ensure systems stability.

In general, the unique aggregation behavior of dimeric surfactants with short spacers significantly influences the rheological behavior of their solutions; i.e., the change of the micelles’ shape from spherical to cylindrical and wormlike micelles affects their viscosity. This phenomenon attracts special attention due to the viscoelastic behavior of such solutions. Viscoelastic solutions of surfactants’ micelles are currently being used extensively as rheological modifiers in consumer products such as paints, detergents, pharmaceuticals, lubricants, and emulsifiers.^58^ Observed changes in micelles’ shape and size in the investigated systems, at very low concentrations, can offer more advantages in enhancing fluid properties compared to the metal-free dimeric precursor. In addition, contrary to metal-free aggregates, metallomicelles can be used as controllable size nanotemplates in the synthesis of metallic nanoparticles.^27,59^ Therefore, they represent a good alternative to existing methodologies for nanoparticle synthesis, which suffer from several drawbacks including nanoparticle agglomeration, high fabrication costs, safety concerns due to the use of toxic solvents, poor yields, and nonuniform particle sizes.^20^

## Conclusions

Inspired by the fact that the introduction of transition metal ions into the surfactants’ structure leads to the development of new and/or advanced properties of materials, a series of novel dimeric metallosurfactants (12–2–12)[MBr_4_], M = Co, Ni, Cu, and Zn, was synthesized in solvated and nonsolvated forms, and investigated both in solution and in the solid state. The obtained results demonstrate that the incorporation and choice of metal more dramatically influence the arrangement of surfactant molecules in the solid state and their magnetic properties, in comparison to their self-assembly features. However, all synthesized metallosurfactants demonstrate lower cmc values compared to the metal-free precursor. In the solid state, (12–2–12)^2+^ cations adopt either V-, U-, or *trans*-conformations, in relation to the spacer plane, depending on the nature of the coordination entity. Incorporation of transition metal ions into the layered, lamellar structures of the dimeric surfactant led to specific magnetic properties. The Co, Ni, and Cu containing compounds behave as low-dimensional antiferromagnetic spin systems in which the antiferromagnetic superexchange interaction between the spins is confined to one or two dimensions. In the 2D magnetic lattice, the relevant super-superexchange interactions are mediated via M–Br-Br-M paths and are confined to a plane. However, the (12–2–12)_2_[NiBr_2_(H_2_O)_4_]Br_4_·2H_2_O compound is most likely a realization of spin *S* = 1 1D Heisenberg antiferromagnet due to Ni–Br–Br–Ni paths being confined to one dimension.

The investigated systems represent a fruitful platform for the development of multifunctional materials, as they are simple to produce, can be obtained in high yields, and show advanced properties both in solution and in the solid state, as compared to their metal-free counterparts. Considering the potential of these systems, future solution studies will be focused on the (i) bactericidal agents with dual action, (ii) synthesis of metallic nanoparticles, i.e., in one molecule investigated surfactants contain metal ions and effective capping agent, and (iii) broad spectrum catalysts. Finally, the title compounds hold great promise for the development of 2D magnetic materials in the solid state, which are currently the focus of research for possible spintronics (electronics based on manipulation of electron spin) applications where the goals are greater read/write density, faster manipulation of states, and high energy efficiency.

## Supporting Information Available

Characterization of synthesized dimeric metallosurfactants (CHN, FTIR, TGA); additional experimental details for single crystal analysis, magnetic and self-assembly properties; table with experimental and crystallographic data; figures with ORTEP plots; Mercury representations of symmetrically nonequivalent (12–2–12)^2+^ cations and the corresponding packing diagrams for (12–2–12)[MBr_4_], M = Co and Zn, and (12–2–12)[NiBr_4_]·CH_3_CN; tables with data for selected bond lengths, angles and hydrogen bond parameters in the crystal structures; figures of the 2D magnetic lattice with shortest Br–Br distances for (12–2–12)[CoBr4]·MeOH and (12–2–12)[NiBr_4_]; plot of temperature dependence of the magnetic spin-only susceptibility multiplied with temperature expressed in emu K/mol of transition metal ions for dimeric metallosurfactants; photos of colored dimeric metallosurfactants in powder form and in solutions, along with the corresponding metallic bromides used in the synthesis; table with measured average hydrodynamic diameters (*d*_h_) of micelles; references for the SI (PDF)
