# Supplementary material for: Transition Metal-Based Dimeric Metallosurfactants: From Organic–Inorganic Hybrid Structures and Low-Dimensional Magnets to Metallomicelles
Source: Inorg Chem. 2024 Jun 17;63(26):12218–30. doi: 10.1021/acs.inorgchem.4c01550 (PMC11220752; doi:10.1021/acs.inorgchem.4c01550)
Supplement: Supplementary file 1 — ic4c01550_si_001.pdf [file ic4c01550_si_001.pdf]

Supporting Information (SI) for:

Transition Metal-Based Dimeric Metallosurfactants: From Organic-Inorganic Hybrid Structures and Low-Dimensional Magnets to Metalomicelles

Mirta Rubčić[a], Mirta Herak[b], Leona Zagorec[c], Darija Domazet Jurašin[d*]

[a]Faculty of Science, Department of Chemistry, University of Zagreb, Horvatovac 102a, HR-10000 Zagreb, Croatia, mirta@chem.pmf.hr

[b]Institute of Physics, Department for Research of Materials Under Extreme Conditions, Bijenička cesta 46, HR-10000 Zagreb, Croatia, mirta@ifs.hr

[c]University of Zagreb, Faculty of Chemical Engineering and Technology, Trg Marka Marulića 19, HR-10000 Zagreb, Croatia, lzagorec@fkit.hr

[d]Ruđer Bošković Institute, Division of Physical Chemistry, Bijenička 54, HR-10000 Zagreb, Croatia, e-mail: darija.jurasin@irb.hr

*corresponding author:

Darija Domazet Jurašin

Division of Physical Chemistry

Ruđer Bošković Institute

Bijenička cesta 54

10 000 Zagreb, Croatia

tel: + 385 1 4561074

+ 385 98 561182

e-mail: darija.jurasin@irb.hr

Characterization of synthesized metallosurfactants. Purity of the samples was checked with **elemental analysis: (12-2-12)[CoBr$_4$]**, blue powder (yield 84 %), $C_{30}H_{66}N_2CoBr_4$, found (calculated): %C 43.44 (43.23); %H 7.88 (8.00), %N 3.45 (3.36), **(12-2-12)[NiBr$_4$]**, blue powder (yield 82 %), $C_{30}H_{66}N_2NiBr_4$, found (calculated): %C 43.16 (43.22); %H 8.91 (7.99), %N 3.49 (3.36), **(12-2-12)[CuBr$_4$]**, dark purple powder (yield 84 %), $C_{30}H_{66}N_2CuBr_4$, found (calculated): %C 43.20 (42.99); %H 7.95 (7.95), %N 3.35 (3.34), **(12-2-12)[ZnBr$_4$]**, white powder (yield 83 %), $C_{30}H_{66}N_2ZnBr_4$, found (calculated): %C 41.78 (42.89); %H 7.05 (7.94), %N 3.15 (3.34).

FTIR ATR (attenuated total reflectance) spectra were recorded on a Nicolet iS50 spectrometer in a 4000–400 cm^{-1} spectral range.

IR (ATR, cm^{-1}): **(12-2-12)[CoBr$_4$]·MeOH**: 3498 br υ(O–H); 2918 υ_a(CH$_2$); 2851 υ_s(CH$_2$); 1477 δ(CH$_3$); 1466 δ(CH$_2$); 1030 υ(C−O), **(12-2-12)[CoBr$_4$]**: 2919 υ_a(CH$_2$); 2851 υ_s(CH$_2$); 1477 δ(CH$_3$); 1466 δ(CH$_2$), **(12-2-12)[NiBr$_4$]·CH$_3$CN**: 3520 br, 3313 br υ(O–H); 2917 υ_a(CH$_2$); 2850 υ_s(CH$_2$); 1617 δ(O–H); 1488 δ(CH$_3$); 1469 δ(CH$_2$), **(12-2-12)$_2$[NiBr$_2$(H$_2$O)$_4$]Br$_4$·2H$_2$O**: 3520 br, 3313 br υ(O–H); 2917 υ_a(CH$_2$); 2850 υ_s(CH$_2$); 1617 δ(O–H); 1488 δ(CH$_3$); 1469 δ(CH$_2$), **(12-2-12)[CuBr$_4$]·MeOH**: 3489 br υ(O–H); 2917 υ_a(CH$_2$); 2850 υ_s(CH$_2$); 1478 δ(CH$_3$); 1466 δ(CH$_2$); 1030 υ(C−O), **(12-2-12)[CuBr$_4$]**: 2920 υ_a(CH$_2$); 2851 υ_s(CH$_2$); 1477 δ(CH$_3$); 1466 δ(CH$_2$), **(12-2-12)[ZnBr$_4$]·MeOH**: 3496 br υ(O–H); 2918 υ_a(CH$_2$); 2851 υ_s(CH$_2$); 1478 δ(CH$_3$); 1466 δ(CH$_2$); 1027 υ(C−O), **(12-2-12)[ZnBr$_4$]**: 2918 υ_a(CH$_2$); 2851 υ_s(CH$_2$); 1477 δ(CH$_3$); 1466 δ(CH$_2$)

Thermogravimetric analyses were conducted on a Mettler-Toledo TGA/DSC 3+ thermobalance with aluminium crucibles under dynamic nitrogen stream of 50 mL min^{-1} in a temperature range between 25 °C and 600 °C, while the heating rate was set to 10 °C·min^{-1}. The results of experiments were processed with the Mettler Toledo STARe Evaluation Software 16.10.

The percentage of solvent mass loss in thermograms quantitatively corresponds to the stoichiometry of the solvates:

(12-2-12)[CoBr$_4$]·MeOH, found (calculated): %CH$_3$OH 3.57 (3.70);

(12-2-12)[CuBr$_4$]·MeOH, found (calculated): %CH$_3$OH 3.72 (3.68);

(12-2-12)[ZnBr$_4$]·MeOH, found (calculated): %CH$_3$OH 4.20 (3.67).

(a)

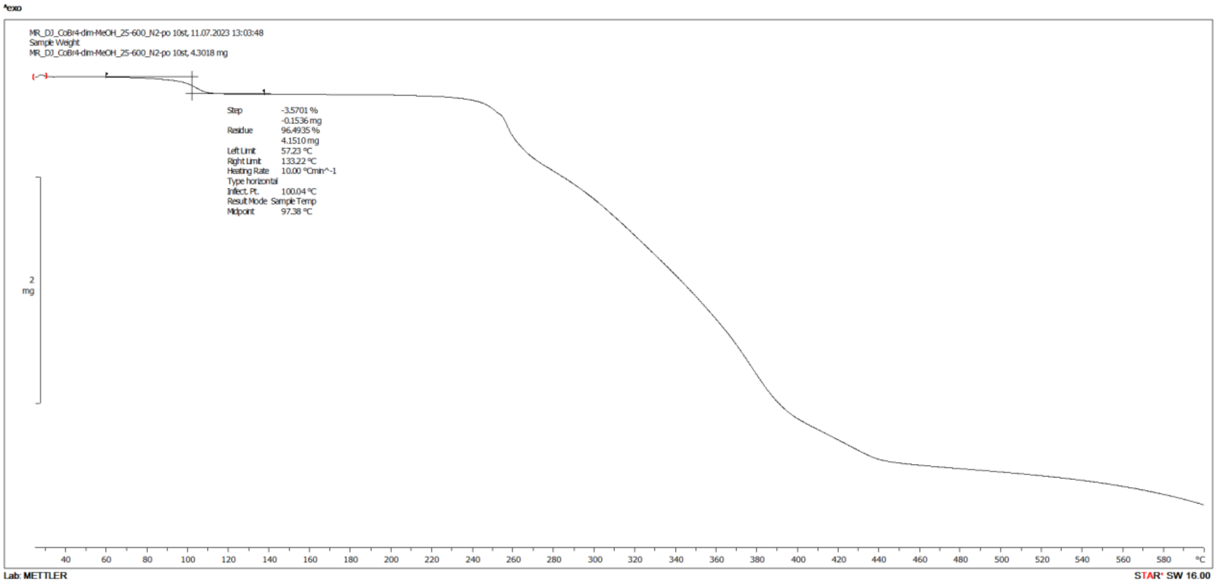

^exo
MR_DJ_CoBr4-dim-MeOH_25-600_N2-po 10st, 11.07.2023 13:03:48
Sample Weight
MR_DJ_CoBr4-dim-MeOH_25-600_N2-po 10st, 4.3018 mg
Step -3.5701 %
-0.1536 mg
Residue 96.4935 %
4.1510 mg
Left Limit 57.23 °C
Right Limit 133.22 °C
Heating Rate 10.00 °Cmin^-1
Type horizontal
Infect. Pt. 100.04 °C
Result Mode Sample Temp
Midpoint 97.38 °C
2
mg
40 60 80 100 120 140 160 180 200 220 240 260 280 300 320 340 360 380 400 420 440 460 480 500 520 540 560 580 °C
Lab: METTLER
STARe SW 16.00

(b)

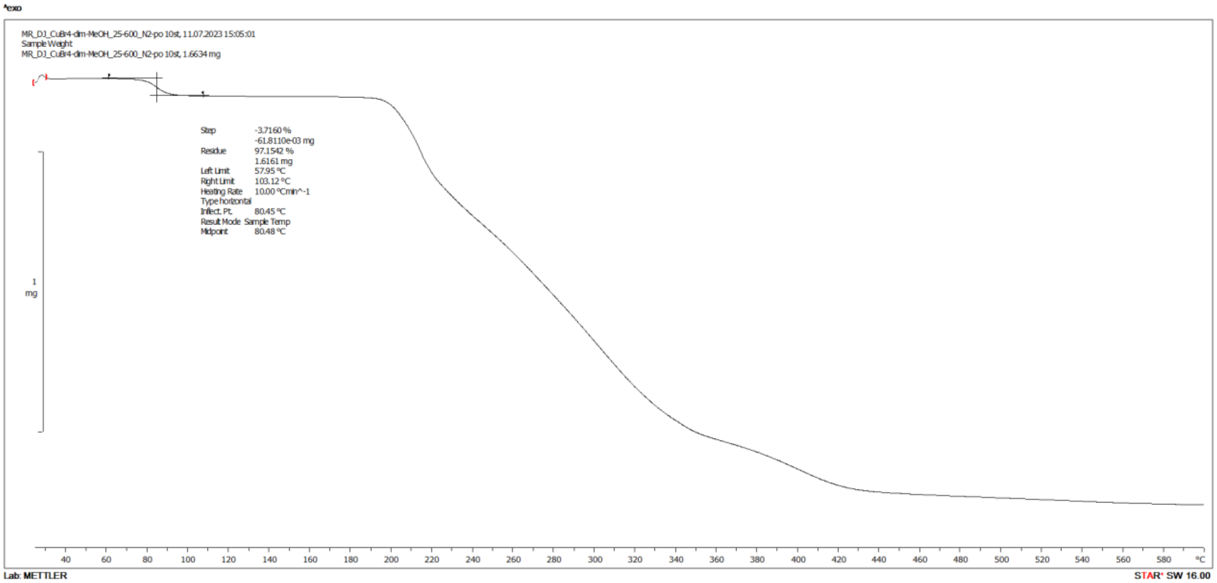

^exo
MR_DJ_CuBr4-dim-MeOH_25-600_N2-po 10st, 11.07.2023 15:05:01
Sample Weight
MR_DJ_CuBr4-dim-MeOH_25-600_N2-po 10st, 1.6634 mg
Step -3.7160 %
-61.8110e-03 mg
Residue 97.1542 %
1.6161 mg
Left Limit 57.95 °C
Right Limit 103.12 °C
Heating Rate 10.00 °Cmin^-1
Type horizontal
Infect. Pt. 80.45 °C
Result Mode Sample Temp
Midpoint 80.48 °C
1
mg
40 60 80 100 120 140 160 180 200 220 240 260 280 300 320 340 360 380 400 420 440 460 480 500 520 540 560 580 °C
Lab: METTLER
STARe SW 16.00

(c)

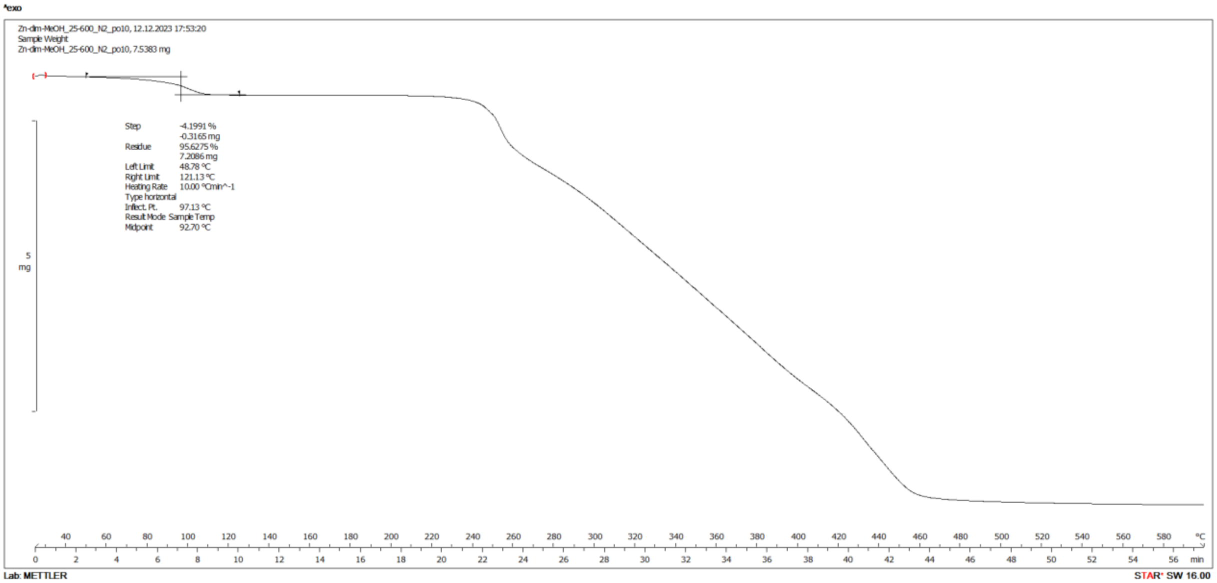

Fig. S1. Thermograms for solvated forms of dimeric metallosurfactants:
(a) (12-2-12)[$CoBr_4$]·MeOH; **(b)** (12-2-12)[$CuBr_4$]·MeOH and **(c)** (12-2-12)[$ZnBr_4$]·MeOH.

Single crystal analysis. The structures were solved with dual space methods using SHELXT [1]. The refinement procedure was done by full-matrix least-squares methods based on F^2 values against all reflections, including anisotropic displacement parameters for all non-H atoms. Hydrogen atoms bound to carbon atoms were placed in geometrically idealized positions and refined by the use of the riding model with $U_{iso} = 1.2U_{eq}$ of the connected carbon atom or as ideal CH_3 groups with $U_{iso} = 1.5U_{eq}$. Hydrogen atoms attached to heteroatoms were located in the difference Fourier maps at the final stages of the refinement procedure. All refinements were performed using SHELXL [2]. The SHELX programs operated within the Olex2 suite [3]. Geometrical calculations were performed by Platon [4] and molecular graphics were done with Mercury [5]. The structure of (12-2-12)[$NiBr_4$]·CH_3CN was solved as non-merohedral two-component twin, with the two components being present in the 0.54:0.46 ratio. The twin law was found to be such that the second twin component was rotated by -179.34° around [0.98 0.00 -0.22] (reciprocal space) or [1.00 0.00 0.00] (direct space).

Data interpretation:

Magnetic properties

Temperature dependence of the measured magnetic susceptibility for all the complexes can be described by expression:

$$\chi\,(T) = \chi_{spin}(T) + \chi_0 \qquad \text{(eq.S1)}$$

where $\chi_{\text{spin}}(T)$ is the temperature-dependent spin-only paramagnetic contribution to the susceptibility and χ_0 is the temperature-independent part of the measured susceptibility. χ_0 includes the temperature-independent contributions from the sample as well as sample holder. In the wide temperature range (50 K $\lesssim T \leq$ 300 K) $\chi_{\text{spin}}(T)$ is described by the Curie-Weiss law given by:

$$\chi_{spin}\,(T) = \frac{C}{T - \Theta_{\text{CW}}} \qquad \text{(eq.S2)}$$

where C is the Curie constant and Θ_{CW} the Curie-Weiss temperature. The Curie constant is given by $C = N_{\text{A}}\,\mu_{\text{eff}}^2 / 3k_{\text{B}}$ where N_{A} is the Avogadro's constant, k_{B} the Boltzmann constant and the effective magnetic moment $\mu_{\text{eff}} = g\mu_{\text{B}}\,[S\,(S+1)]^{1/2}$, where g is the electron g factor, μ_{B} the Bohr magneton and S the spin of the transition metal ion. The Curie-Weiss temperature Θ_{CW}, expressed in Kelvin, corresponds to the effective magnetic interactions between the magnetic moments, where positive value of Θ_{CW} reflects the ferromagnetic interaction, and negative value the antiferromagnetic interaction [6].

Self-assembly properties

Values of the critical micelle concentration (cmc) were determined from the intersection of the two straight lines drawn at low and high concentration regions in the surface tension (γ) and the electrical conductivity (κ) *vs.* surfactant concentration c((12-2-12)[MBr_4]) curves using a linear regression analysis.

Table S1. Experimental and crystallographic data for dimeric metallosurfactants in this work:

**Identifier**	**(12-2-12)[CuBr$_4$]**	**(12-2-12)[CuBr$_4$]·MeOH**	**(12-2-12)[CoBr$_4$]**	**(12-2-12)[CoBr$_4$]·MeOH**	**(12-2-12)[ZnBr$_4$]**	**(12-2-12)[NiBr$_4$]·CH$_3$CN**	**(12-2-12)$_2$[NiBr$_2$(H$_2$O)$_4$]Br$_4$·2H$_2$O**
Empirical formula	$C_{30}H_{66}Br_4CuN_2$	$C_{31}H_{70}Br_4CuN_2O$	$C_{30}H_{66}Br_4CoN_2$	$C_{31}H_{70}Br_4CoN_2O$	$C_{30}H_{66}Br_4ZnN_2$	$C_{32}H_{69}Br_4N_3Ni$	$C_{60}H_{144}Br_6N_4NiO_6$
M_r	838.02	870.04	833.41	865.46	839.85	874.19	1555.88
T/K	170(2)	170(2)	170(2)	170(2)	170(2)	170(2)	293(2)
Crystal system	triclinic	triclinic	triclinic	monoclinic	triclinic	triclinic	triclinic
Space group	P-1	P-1	P-1	$P2_1/c$	P-1	P-1	P-1
a/Å	8.0407(2)	14.44715(11)	8.1473(2)	15.2066(3)	8.1460(2)	14.6966(2)	8.11942(5)
b/Å	14.2990(3)	14.85623(9)	14.5402(4)	36.8522(6)	14.5505(3)	15.1663(2)	9.60429(6)
c/Å	18.3253(5)	19.13865(11)	18.0771(5)	14.3119(2)	18.0641(6)	19.3868(4)	26.90406(19)
α/°	67.136(2)	100.6178(5)	66.568(3)	90	66.530(2)	107.056(2)	96.9367(5)
β/°	86.525(2)	97.9222(6)	82.888(2)	90.103(2)	82.934(2)	99.7680(10)	97.1890(5)
γ/°	75.148(2)	96.2549(6)	77.332(2)	90	77.372(2)	89.9950(10)	101.4239(5)
V/Å^3	1874.73(9)	3960.60(5)	1915.44(10)	8020.3(2)	1914.92(9)	4065.58(12)	2017.30(2)
Z	2	4	2	8	2	4	1
ρ_{calc}/g cm^{-3}	1.485	1.459	1.445	1.433	1.457	1.428	1.281
μ/mm^{-1}	5.948	5.671	8.529	8.187	5.909	5.451	4.142
F(000)	858.0	1788.0	854.0	3560.0	860.0	1800.0	818.0
Crystal size/mm^3	0.17 × 0.16 × 0.06	0.25 × 0.1 × 0.03	0.21 × 0.15 × 0.04	0.19 × 0.12 × 0.02	0.22 × 0.09 × 0.03	0.18 × 0.15 × 0.01	0.22 × 0.16 × 0.09
Radiation	CuKα (λ = 1.54184)						
2Θ range/°	5.238 to 156.362	6.112 to 159.914	5.332 to 134.99	6.626 to 133.986	5.338 to 161.996	4.844 to 135	6.698 to 159.792

Index ranges	-10 ≤ h ≤ 10, -17 ≤ k ≤ 14, -23 ≤ l ≤ 23	-16 ≤ h ≤ 18, -17 ≤ k ≤ 18, -24 ≤ l ≤ 24	-9 ≤ h ≤ 9, -17 ≤ k ≤ 17, -21 ≤ l ≤ 21	-16 ≤ h ≤ 18, -43 ≤ k ≤ 43, -17 ≤ l ≤ 17	-10 ≤ h ≤ 10, -13 ≤ k ≤ 18, -23 ≤ l ≤ 22	-17 ≤ h ≤ 17, -18 ≤ k ≤ 18, -23 ≤ l ≤ 23	-10 ≤ h ≤ 10, -12 ≤ k ≤ 12, -34 ≤ l ≤ 31
Reflections collected	34206	77475	56110	155742	31866	25225	75751
Independent reflections	7836 [R_{int} = 0.0498, R_{sigma} = 0.0311]	16961 [R_{int} = 0.0454, R_{sigma} = 0.0331]	6889 [R_{int} = 0.1040, R_{sigma} = 0.0374]	14141 [R_{int} = 0.1435, R_{sigma} = 0.0475]	8197 [R_{int} = 0.0592, R_{sigma} = 0.0412]	25225 [R_{int} = -, R_{sigma} = 0.0260]	8696 [R_{int} = 0.0296, R_{sigma} = 0.0133]
Data/restraints/parameters	7836/0/340	16961/0/718	6889/0/340	14141/1/719	8197/0/340	25225/0/736	8696/9/379
Goodness–of–fit on F^2, S^b	1.114	1.060	1.124	1.125	1.053	1.014	1.041
Final R and wR^c values [I≥2σ(I)]	R_1 = 0.0948, wR_2 = 0.2585	R_1 = 0.0359, wR_2 = 0.1015	R_1 = 0.0630, wR_2 = 0.1618	R_1 = 0.0874, wR_2 = 0.2743	R_1 = 0.0559, wR_2 = 0.1532	R_1 = 0.0750, wR_2 = 0.2070	R_1 = 0.0304, wR_2 = 0.0900
Final R and wR^c values [all data]	R_1 = 0.0991, wR_2 = 0.2605	R_1 = 0.0376, wR_2 = 0.1032	R_1 = 0.0710, wR_2 = 0.1673	R_1 = 0.1174, wR_2 = 0.2992	R_1 = 0.0637, wR_2 = 0.1586	R_1 = 0.0841, wR_2 = 0.2177	R_1 = 0.0316, wR_2 = 0.0915
Largest diff. peak/hole / e Å^{-3}	2.52/-1.34	1.92/-0.89	1.51/-1.28	1.14/-1.97	1.38/-1.54	1.31/-0.93	1.65/-0.50

[a] $w = 1/[\sigma F_o^2 + (g_1P)^2 + g_2P]$ where $P = (F_o^2 + 2F_c^2)/3$

[b] $S = \{\Sigma[w(F_o^2 - F_c^2)2]/(N_r - N_p)\}^{1/2}$ where N_r = number of independent reflections, N_p = number of refined parameters.

[c] $R = \Sigma||F_o| - |F_c|/\Sigma|F_o|$; $wR = \{\Sigma[w(F_o^2 - F_c^2)2]/\Sigma[w(F_o^2)2]\}^{1/2}$

(a)

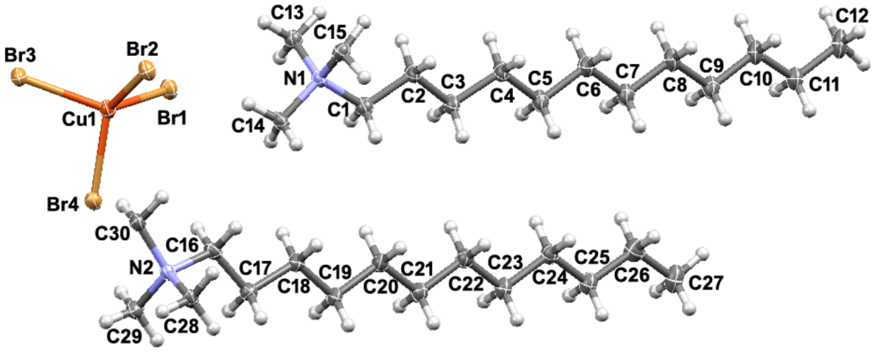
Br3
Br2
Br1
Cu1
Br4
C13
C15
N1
C14
C1
C2
C3
C4
C5
C6
C7
C8
C9
C10
C11
C12
C30
C16
N2
C29
C28
C17
C18
C19
C20
C21
C22
C23
C24
C25
C26
C27

(b)

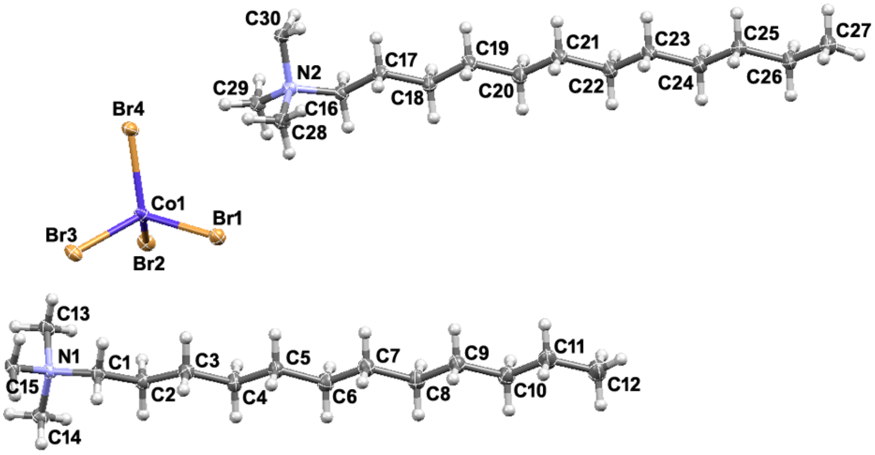
C30
C29
N2
C16
C28
C17
C18
C19
C20
C21
C22
C23
C24
C25
C26
C27
Br4
Co1
Br1
Br3
Br2
C13
N1
C15
C14
C1
C2
C3
C4
C5
C6
C7
C8
C9
C10
C11
C12

(c)

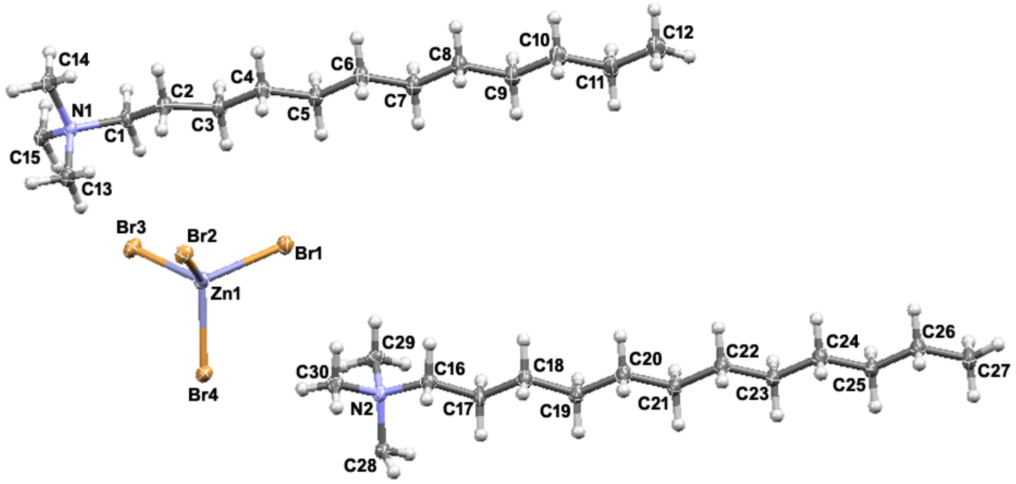
C14
N1
C15
C13
C1
C2
C3
C4
C5
C6
C7
C8
C9
C10
C11
C12
Br3
Br2
Br1
Zn1
Br4
C29
C30
N2
C28
C16
C17
C18
C19
C20
C21
C22
C23
C24
C25
C26
C27

(d)

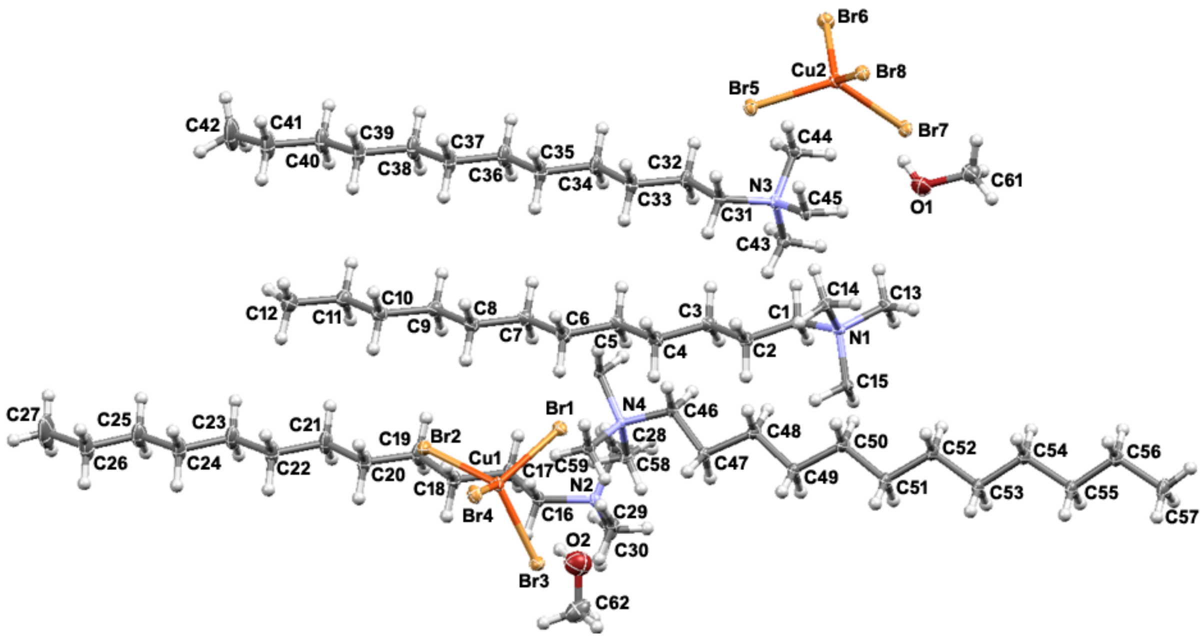
Br6
Cu2
Br8
Br5
Br7
C42
C41
C39
C37
C35
C32
C40
C38
C36
C34
C33
C31
N3
C44
C45
C43
O1
C61
C12
C11
C10
C9
C8
C7
C6
C5
C4
C3
C2
C1
C14
C13
N1
C15
C27
C25
C26
C23
C24
C21
C22
C19
C20
Br2
C18
Cu1
Br1
Br4
C17
C59
N4
C28
C58
C46
C47
C48
C49
C50
C51
C52
C53
C54
C55
C56
C57
N2
C16
C29
C30
O2
Br3
C62

(e)

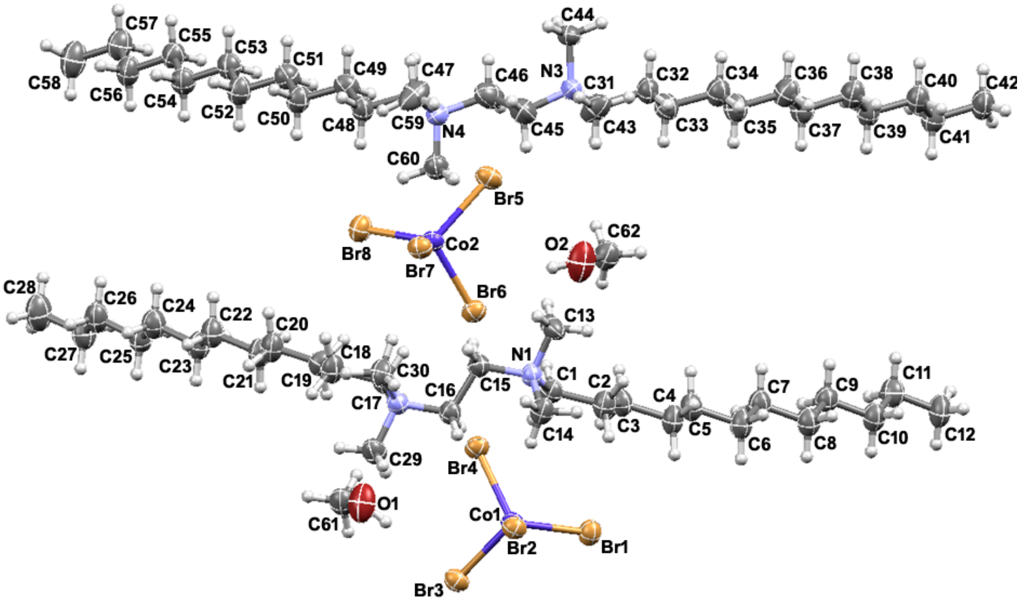
C57
C55
C53
C51
C49
C47
C46
N3
C44
C31
C32
C34
C36
C38
C40
C42
C58
C56
C54
C52
C50
C48
C59
N4
C45
C43
C33
C35
C37
C39
C41
C60
Br5
Br8
Co2
Br7
Br6
O2
C62
C28
C26
C24
C22
C20
C18
C27
C25
C23
C21
C19
C30
C16
C15
N1
C1
C13
C2
C17
C3
C4
C5
C6
C7
C8
C9
C10
C11
C12
C14
C29
Br4
O1
C61
Co1
Br2
Br1
Br3

(f)

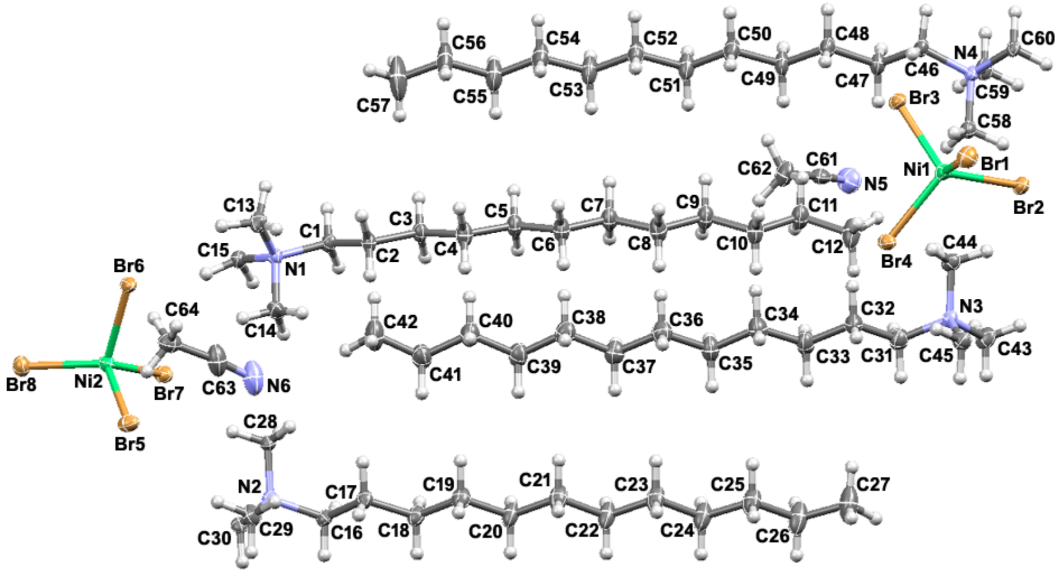

(g)

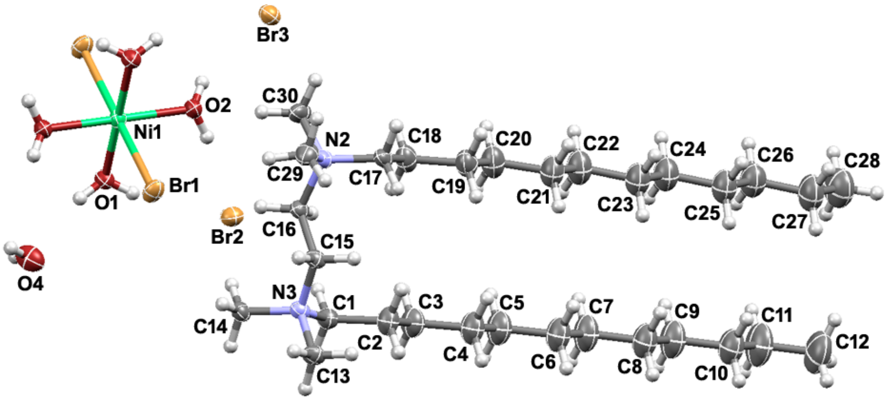

Fig. S2. Asymmetric units of: **(a)** (12-2-12)[$CuBr_4$], **(b)** (12-2-12)[$CoBr_4$], **(c)** (12-2-12)[$ZnBr_4$], **(d)** (12-2-12)[$CuBr_4$]·MeOH, **(e)** (12-2-12)[$CoBr_4$]·MeOH, and **(f)** (12-2-12) [$NiBr_4$]·CH_3CN with the atom numbering schemes. **(g)** Molecular structure of $(12\text{-}2\text{-}12)_2[NiBr_2(H_2O)_4]Br_4 \cdot 2H_2O$, with the corresponding atom numbering scheme, where only the atoms that belong to asymmetric unit are numbered. In (a)-(g) ellipsoids are drawn at 30% probability level, while hydrogen atoms are drawn as spheres of arbitrary radii.

Table S2. Selected bond lengths, angles and hydrogen bond parameters in the crystal structure of (12-2-12)[$CuBr_4$].

Atoms	Bond length/Å	Atoms	Bond angle/°	Atoms	Bond angle/°
Br1–Cu1	2.373(2)	Br3–Cu1–Br4	101.97(8)	Br2–Cu1–Br3	101.08(7)
Br2–Cu1	2.401(2)	Br1–Cu1–Br4	100.12(7)	Br2–Cu1–Br4	127.73(8)
Br3–Cu1	2.400(2)	Br1–Cu1–Br2	102.78(8)		
Br4–Cu1	2.379(2)	Br1–Cu1–Br3	126.24(8)		
τ_4 value*	0.75				

* $\tau_4 = \frac{360° - (\alpha+\beta)}{141°}$; α and β are two largest angles in the four-coordinate species.[7]

D–H···A	D-H	H···A	D···A	∠D–H···A	Symmetry code
C1–H1A···Br1	0.9900	2.9000	3.785(12)	149.00	-1+x,y,z
C13–H13A···Br3	0.9800	2.8800	3.793(12)	156.00	2-x,1-y,2-z
C14–H14B···Br2	0.9800	2.7700	3.729(12)	166.00	.
C15–H15B···Br1	0.9900	2.8800	3.814(11)	157.00	-1+x,y,z
C28–H28B···Br2	0.9800	2.8900	3.795(12)	154.00	1-x,2-y,2-z
C29–H29A···Br4	0.9800	2.9100	3.821(13)	154.00	.
C30–H30A···Br3	0.9900	2.9100	3.827(12)	155.00	-1+x,y,z
C30–H30B···Br4	0.9900	2.8800	3.796(11)	155.00	.

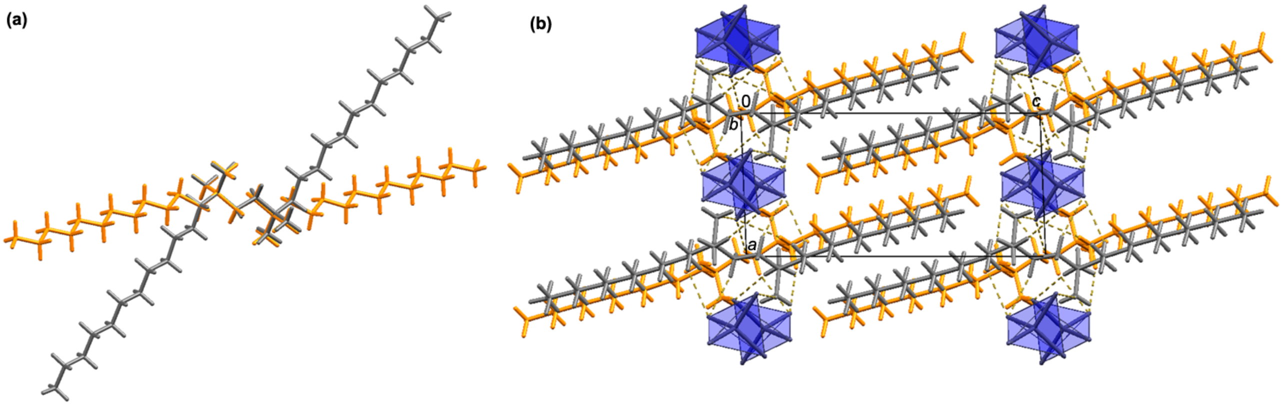

Fig. S3. (a) Overlay diagram of the two symmetrically non-equivalent $(12\text{-}2\text{-}12)^{2+}$ cations (shown in gray-N2 containing cation and orange-N1 containing cation) in (12-2-12)[$CoBr_4$]. Crystal packing in (12-2-12)[$CoBr_4$] shown down the: (b) *b*-axis. In (b) $[CoBr_4]^{2-}$ anions are presented in polyhedral style.

Table S3. Selected bond lengths, angles and hydrogen bond parameters in the crystal structure of (12-2-12)[$CoBr_4$].

Atoms	Bond length/Å	Atoms	Bond angle/°	Atoms	Bond angle/°
Br1–Co1	2.4124(14)	Br3–Co1–Br4	106.43(5)	Br2–Co1–Br3	107.39(5)
Br2–Co1	2.4174(14)	Br1–Co1–Br4	108.95(5)	Br2–Co1–Br4	114.70(5)
Br3–Co1	2.4238(12)	Br1–Co1–Br2	106.85(5)		
Br4–Co1	2.4187(13)	Br1–Co1–Br3	112.64(5)		
τ_4 value*	0.94				

* $\tau_4 = \frac{360° - (\alpha+\beta)}{141°}$; α and β are two largest angles in the four-coordinate species.[7]

D–H···A	D-H	H···A	D···A	∠D–H···A	Symmetry code
C1–H1B···Br3	0.9700	2.8800	3.791(7)	156.00	-
C13–H13A···Br4	0.9600	2.9300	3.803(8)	152.00	1+x,y,z
C15–H15A···Br2	0.9700	2.7900	3.708(7)	157.00	1+x,y,z
C15–H15B···Br3	0.9700	2.9100	3.804(7)	154.00	-
C28–H28C···Br3	0.9600	2.8500	3.754(9)	158.00	1+x,y,z
C29–H29B···Br4	0.9600	2.7600	3.701(7)	165.00	-
C30–H30B···Br1	0.9700	2.8400	3.742(7)	154.00	-1+x,y,z

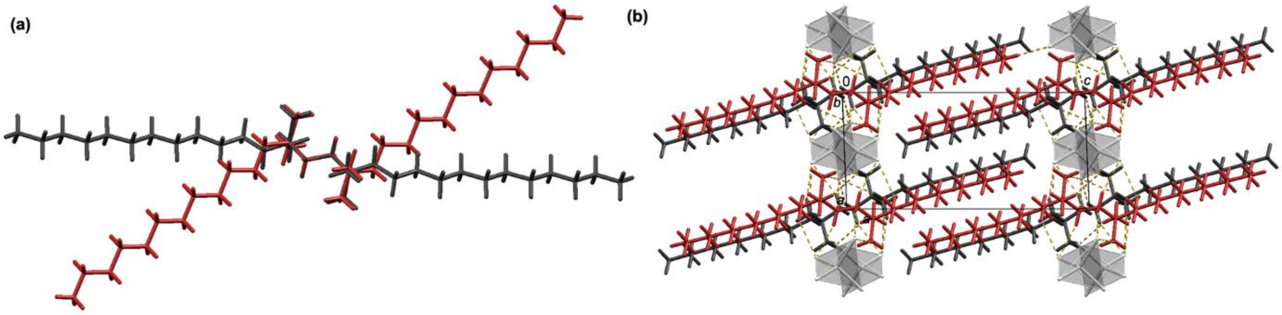

Fig. S4. (a) Overlay diagram of the two symmetrically non-equivalent $(12\text{-}2\text{-}12)^{2+}$ cations (red-N2 containing cation and gray-N1 containing cation) in $[ZnBr_4](12\text{-}2\text{-}12)$. Crystal packing in $[ZnBr_4](12\text{-}2\text{-}12)$ shown down the: (b) *b*-axis. In (b) $[ZnBr_4]^{2-}$ anions are presented in polyhedral style.

Table S4. Selected bond lengths, angles and hydrogen bond parameters in the crystal structure of $(12\text{-}2\text{-}12)[ZnBr_4]$.

Atoms	Bond length/Å	Atoms	Bond angle/°	Atoms	Bond angle/°
Br1–Zn1	2.4123(8)	Br3–Zn1–Br4	106.50(3)	Br2–Zn1–Br3	107.43(3)
Br2–Zn1	2.4163(9)	Br1–Zn1–Br4	109.17(3)	Br2–Zn1–Br4	114.45(3)
Br3–Zn1	2.4285(8)	Br1–Zn1–Br2	107.18(3)		
Br4–Zn1	2.4177(8)	Br1–Zn1–Br3	112.21(3)		
τ_4 value*	0.95				

* $\tau_4 = \frac{360° - (\alpha+\beta)}{141°}$; α and β are two largest angles in the four-coordinate species.[7]

D–H···A	D-H	H···A	D···A	∠D–H···A	Symmetry code
C1–H1B···Br3	0.9900	2.8500	3.777(5)	156.00	-
C13–H13A···Br4	0.9800	2.9000	3.803(5)	154.00	1-x,1-y,-z
C15–H15A···Br2	0.9900	2.7800	3.710(4)	157.00	1+x,y,z
C15–H15B···Br3	0.9900	2.8700	3.790(4)	155.00	-
C28–H28B···Br1	0.9900	2.8300	3.739(4)	154.00	-1+x,y,z
C29–H29C···Br3	0.9800	2.8400	3.747(6)	155.00	1-x,-y,-z
C30–H30B···Br4	0.9800	2.7600	3.702(5)	161.00	-

Table S5. Selected bond lengths, angles and hydrogen bond parameters in the crystal structure of (12-2-12)[$CuBr_4$]·MeOH.

Atoms	Bond length/Å	Atoms	Bond angle/°	Atoms	Bond angle/°
Br1–Cu1	2.4024(4)	Br3–Cu1–Br4	97.05(2)	Br6–Cu2–Br7	101.65(2)
Br2–Cu1	2.3600(5)	Br1–Cu1–Br4	127.78(2)	Br5–Cu2–Br7	128.63(2)
Br3–Cu1	2.3861(5)	Br1–Cu1–Br2	99.65(2)	Br7–Cu2–Br8	97.51(2)
Br4–Cu1	2.3833(5)	Br1–Cu1–Br3	101.59(2)	Br6–Cu2–Br8	131.80(2)
Br5–Cu2	2.3929(4)	Br2–Cu1–Br3	133.13(2)		
Br6–Cu2	2.3578(5)	Br2–Cu1–Br4	102.01(2)		
Br7–Cu2	2.3829(5)	Br5–Cu2–Br6	100.87(2)		
Br8–Cu2	2.3976(5)	Br5–Cu2–Br8	100.70(2)		
τ_4 value(Cu1)*	0.70				
τ_4 value(Cu2)*	0.71				

* $\tau_4 = \frac{360° - (\alpha+\beta)}{141°}$; α and β are two largest angles in the four-coordinate species.[7]

D–H···A	D-H	H···A	D···A	∠D–H···A	Symmetry code
O1–H1 ···Br8	0.8400	2.6000	3.408(3)	162.00	.
O2–H2 ···Br4	0.8400	2.7400	3.458(4)	145.00	1+x,y,z
C13–H13B···Br3	0.9800	2.7600	3.715(2)	164.00	.
C14–H14C···Br8	0.9800	2.9000	3.847(2)	162.00	.
C15–H15A···Br5	0.9900	2.8500	3.788(2)	159.00	-x,2-y,1-z
C29–H29A···Br3	0.9800	2.8600	3.802(3)	161.00	-x,2-y,1-z
C30–H30A···Br8	0.9900	2.8200	3.786(2)	165.00	1-x,2-y,1-z
C30–H30B···Br1	0.9900	2.7300	3.677(2)	159.00	1+x,y,z
C45–H45A···Br5	0.9900	2.9200	3.872(2)	162.00	-x,1-y,1-z
C45–H45B···Br4	0.9900	2.8500	3.767(2)	155.00	.
C60–H60A···Br7	0.9900	2.8400	3.7796(19)	159.00	.
C60–H60B···Br1	0.9900	2.8100	3.751(2)	160.00	-x,1-y,1-z

Table S6. Selected bond lengths, angles and hydrogen bond parameters in the crystal structure of (12-2-12)[$CoBr_4$]·MeOH.

Atoms	Bond length/Å	Atoms	Bond angle/°	Atoms	Bond angle/°
Br1–Co1	2.3934(18)	Br3–Co1–Br4	103.10(6)	Br6–Co2–Br7	108.42(6)
Br2–Co1	2.4140(15)	Br1–Co1–Br4	112.13(6)	Br5–Co2–Br7	109.12(6)
Br3–Co1	2.4161(17)	Br1–Co1–Br2	111.08(6)	Br7–Co2–Br8	112.16(7)
Br4–Co1	2.4112(17)	Br1–Co1–Br3	111.99(6)	Br6–Co2–Br8	112.12(7)
Br5–Co2	2.4151(17)	Br2–Co1–Br3	109.45(6)		
Br6–Co2	2.4067(17)	Br2–Co1–Br4	108.77(6)		
Br7–Co2	2.4164(15)	Br5–Co2–Br6	103.55(6)		
Br8–Co2	2.3893(18)	Br5–Co2–Br8	111.08(7)		
τ_4 value(Co1)*	0.96				
τ_4 value(Co2)*	0.96				

* $\tau_4 = \frac{360° - (\alpha+\beta)}{141°}$; α and β are two largest angles in the four-coordinate species.[7]

D–H···A	D-H	H···A	D···A	∠D–H···A	Symmetry code
O1–H1···Br3	0.8400	2.7000	3.515(9)	163.00	
O2–H2···Br6	0.8400	2.7200	3.525(9)	162.00	
C1–H1B···Br4	0.9900	2.9300	3.719(8)	138.00	
C15–H15B···Br6	0.9900	2.8100	3.731(10)	155.00	*x,3/2-y,-1/2+z*
C16–H16A···Br4	0.9900	2.9000	3.857(9)	162.00	-
C16–H16B···Br4	0.9900	2.8400	3.740(9)	151.00	x,3/2-y,-1/2+z
C45–H45B···Br3	0.9900	2.8300	3.771(12)	158.00	1+x,y,z
C46–H46A···Br5	0.9900	2.7800	3.735(12)	162.00	x,3/2-y,1/2+z
C46–H46B···Br2	0.9900	2.8900	3.844(12)	163.00	1+x,3/2-y,1/2+z

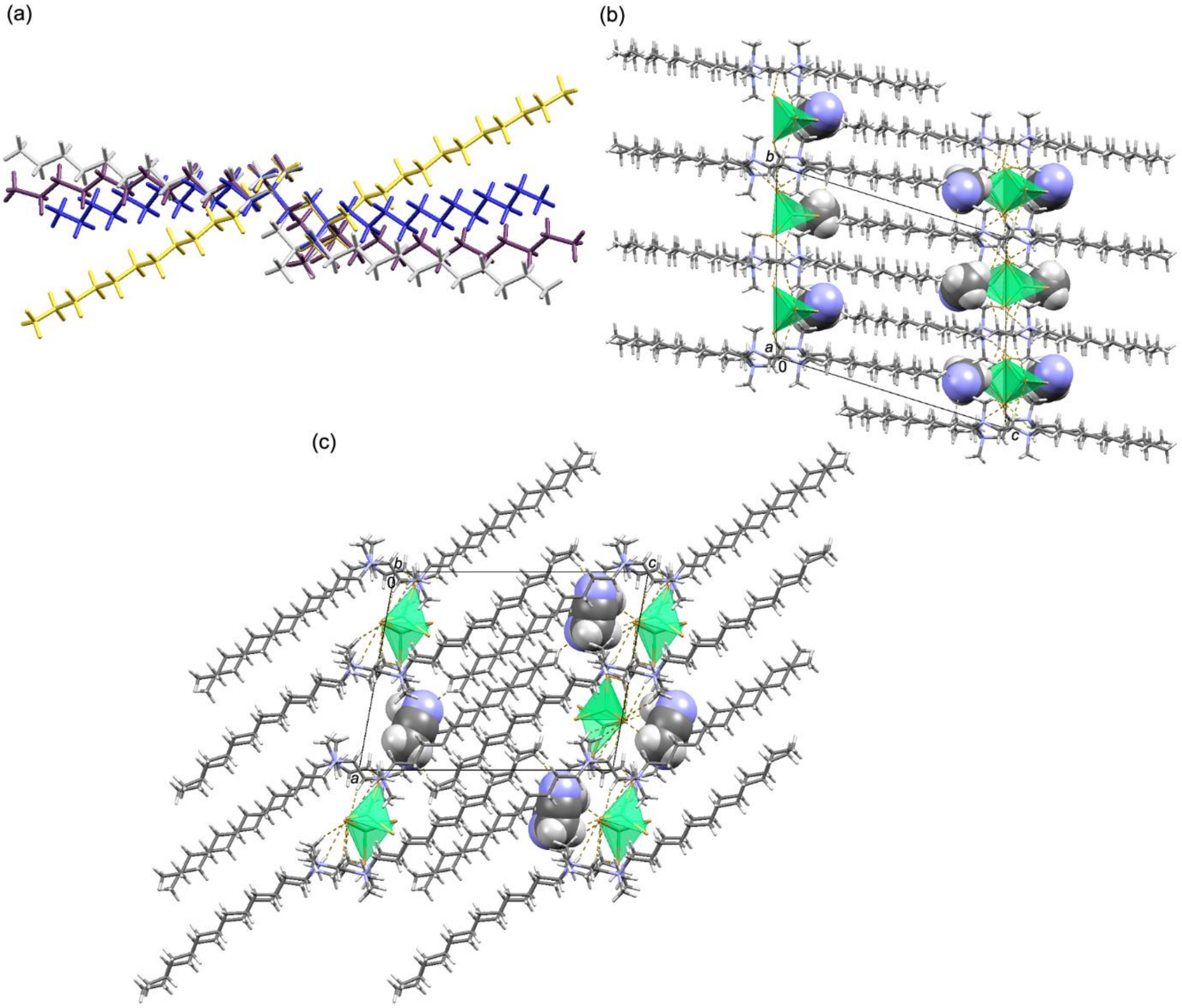

Fig. S5. (a) Overlay diagram of the four symmetrically non-equivalent $(12\text{-}2\text{-}12)^{2+}$ cations (purple-N1, blue-N2, yellow-N3 and gray-N4) in (12-2-12)[$NiBr_4$]·CH_3CN. Crystal packing in (12-2-12)[$NiBr_4$]·CH_3CN shown down the: (b) *a*-axis, (c) *b*-axis. In (b) $[NiBr_4]^{2-}$ anions are presented in polyhedral style, while the acetonitrile molecules are presented in the spacefill style. C–H···Br and C–H···N interactions are highlighted by yellow dashed lines.

Table S7. Selected bond lengths, angles and hydrogen bond parameters in the crystal structure of (12-2-12)$[NiBr_4]\cdot CH_3CN$.

Atoms	Bond length/Å	Atoms	Bond angle/°	Atoms	Bond angle/°
Br1–Ni1	2.3769(15)	Br3–Ni1–Br4	103.08(5)	Br6–Ni2–Br7	106.58(5)
Br2–Ni1	2.4132(14)	Br1–Ni1–Br4	117.07(6)	Br5–Ni2–Br7	108.09(5)
Br3–Ni1	2.3854(14)	Br1–Ni1–Br2	105.79(5)	Br7–Ni2–Br8	109.45(5)
Br4–Ni1	2.4037(14)	Br1–Ni1–Br3	110.29(6)	Br6–Ni2–Br8	113.53(6)
Br5–Ni2	2.3951(15)	Br2–Ni1–Br3	111.49(5)		
Br6–Ni2	2.3956(14)	Br2–Ni1–Br4	109.23(5)		
Br7–Ni2	2.4117(14)	Br5–Ni2–Br6	102.15(5)		
Br8–Ni2	2.3773(14)	Br5–Ni2–Br8	116.39(6)		
τ_4 value(Ni1)*	0.93				
τ_4 value(Ni2)*	0.92				

* $\tau_4 = \frac{360° - (\alpha + \beta)}{141°}$; α and β are two largest angles in the four-coordinate species.[7]

D–H···A	D-H	H···A	D···A	∠D–H···A	Symmetry code
C12–H12B···N5	0.9800	2.5100	3.455(14)	162.00	-
C13–H13A···Br6	0.9800	2.9200	3.819(9)	153.00	-
C15–H15B···Br6	0.9900	2.9000	3.796(8)	151.00	-
C30–H30A···Br7	0.9900	2.8700	3.825(8)	163.00	1-x,1-y,-z
C30–H30B···Br4	0.9900	2.8900	3.826(8)	159.00	x,y,-1+z
C42–H42A···N6	0.9800	2.5600	3.429(17)	147.00	-
C43–H43C···Br5	0.9800	2.6700	3.631(9)	165.00	-1+x,y,1+z
C45–H45A···Br2	0.9900	2.8700	3.835(8)	165.00	-x,1-y,2-z
C60–H60A···Br6	0.9900	2.8900	3.808(6)	155.00	-1+x,y,1+z
C60–H60B···Br2	0.9900	2.8600	3.832(6)	167.00	-x,-y,2-z
C64–H64A···Br6	0.9800	2.8800	3.775(9)	151.00	-

Table S8. Selected bond lengths, angles and hydrogen bond parameters in the crystal structure of $(12\text{-}2\text{-}12)_2[NiBr_2(H_2O)_4]Br_4{\cdot}2H_2O$.

Atoms	Bond length/Å	Atoms	Bond angle/°	Atoms	Bond angle/°
Br1–Ni1	2.5568(2)	Br1^{i}–Ni1–O2^{i}	89.37(4)	Br1^{i}–Ni1–O1	91.70(4)
Ni1–O1	2.1220(15)	O1^{i} –Ni1–O2^{i}	89.16(6)	O1 –Ni1–O1^{i}	180.00
Ni1–O2	2.0371(13)	Br1–Ni1–O1	88.30(4)	O1 –Ni1–O2^{i}	90.84(6)
Ni1–O2^{i}	2.0371(13)	Br1–Ni1–O2	89.37(4)	Br1^{i}–Ni1–O2	90.63(4)
Ni1–O1^{i}	2.1220(15)	Br1–Ni1–Br1^{i}	180.00	O1^{i} –Ni1–O2	90.84(6)
		Br1–Ni1–O1^{i}	91.70(4)	O2 –Ni1–O2_i	180.00
		Br1–Ni1–O2^{i}	90.63(4)	Br1^{i}–Ni1–O1^{i}	88.30(4)
		O1 –Ni1–O2	89.16(6)		

i = 1-x,1-y,2-z

D–H···A	D-H	H···A	D···A	∠D–H···A	Symmetry code
O1–H1C···Br2	0.79(3)	2.64(3)	3.4088(16)	166(3)	-
O1–H1D···O4	0.82(4)	1.98(4)	2.785(3)	166(3)	-
O2–H2C···Br2	0.84(2)	2.40(2)	3.2275(14)	168(2)	-
O2–H2D···Br3	0.83(2)	2.52(2)	3.2817(14)	153(2)	-
O4–H4C···O1	0.84(5)	2.17(5)	2.984(3)	163(4)	2-x,1-y,2-z
O4–H4D···Br3	0.81(2)	2.57(3)	3.361(2)	167(4)	1-x,1-y,2-z
C15–H15A···Br3	0.9700	2.9200	3.6374(18)	131.00	x,1+y,z
C15–H15B···Br1	0.9700	2.8300	3.745(2)	158.00	1-x,2-y,2-z
C15–H15A···Br3	0.9700	2.9200	3.6374(18)	131.00	x,1+y,z
C16–H16B···Br1	0.9700	2.7600	3.721(2)	169.00	-
C17–H17A···Br2	0.9700	2.8100	3.7055(18)	154.00	-1+x,y,z
C30–H30C···O2	0.9600	2.5000	3.164(3)	126.00	-
C16–H16B···Br1	0.9700	2.7600	3.721(2)	169.00	-

(a) (b)

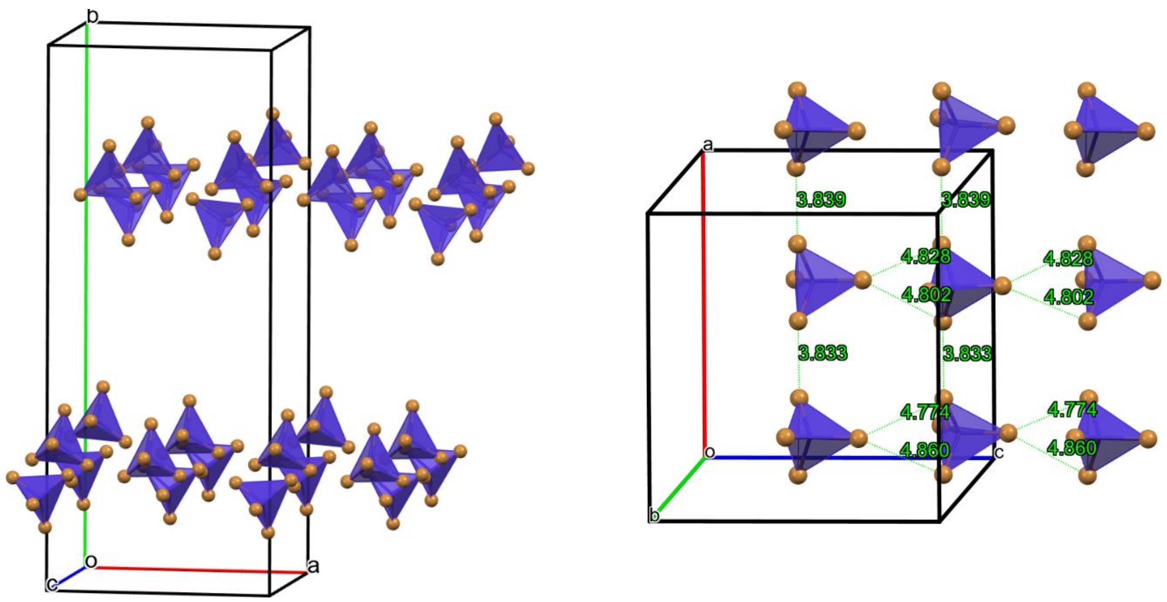

Fig. S6. (a) 2D magnetic lattice in (12-2-12)[CoBr4] · MeOH. Blue tetrahedra represent $[CoBr_4]^{2-}$ anions. The superexchange interaction is expected to run along Co-Br-Br-Co paths. **(b)** The shortest Br-Br distance within the 2D layers. Organic $(12\text{-}2\text{-}12)^{2+}$ cations (not shown for clarity) effectively disable the magnetic interactions along the *b* axis making the system a 2D antiferromagnet with 2D planes confined to the crystallographic *ac* plane.

(a) (b)

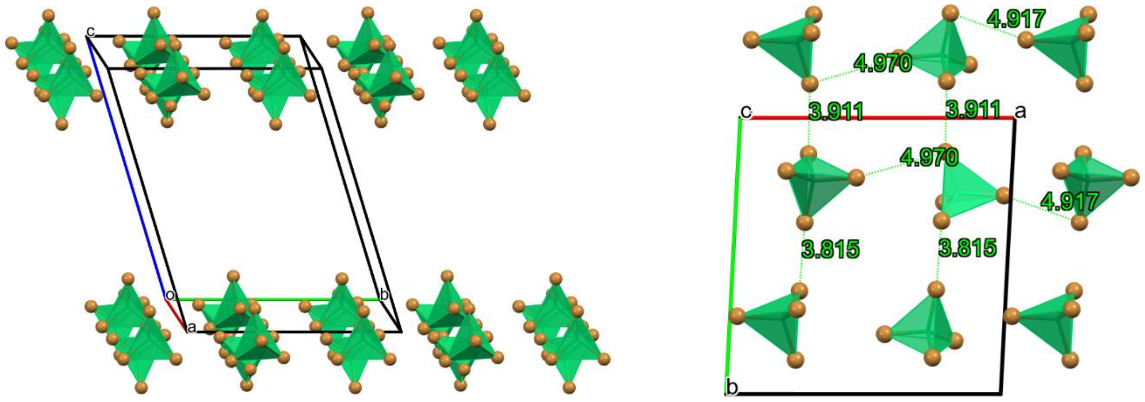

Fig. S7. (a) 2D magnetic lattice in (12-2-12)[$NiBr_4$]. Green tetrahedra represent $[NiBr_4]^{2-}$ anions. The super-superexchange interaction is expected to run along Ni-Br-Br-Ni paths. **(b)** The shortest Br-Br distance within the 2D layers. Organic $(12\text{-}2\text{-}12)^{2+}$ cations (not shown for clarity) effectively disable the magnetic interactions along the c axis making the system a 2D antiferromagnet with 2D planes confined to the crystallographic *ab* plane.

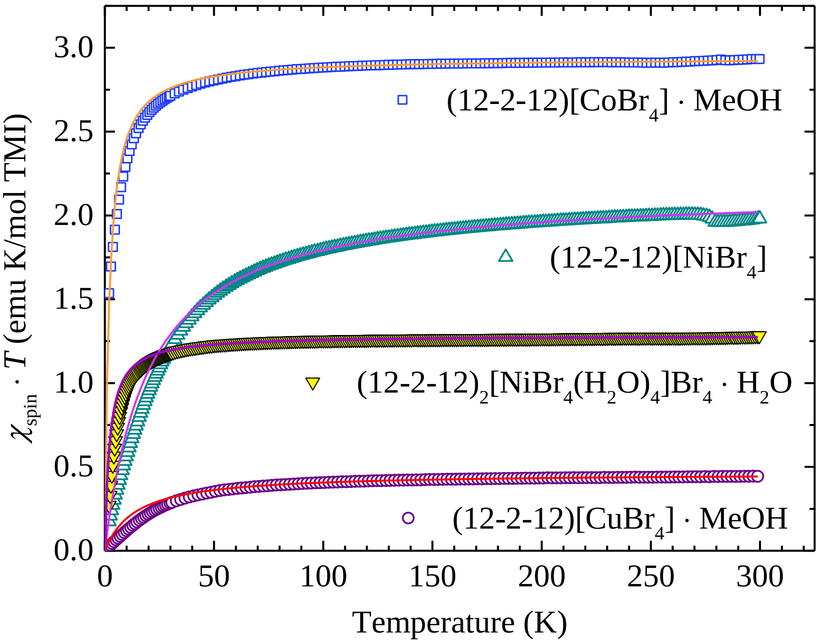

Fig. S8. Temperature dependence of the magnetic spin-only susceptibility multiplied with temperature expressed in emu K/mol of transition metal ions for dimeric metallosurfactants. Solid lines represent the fit of the data to the Curie-Weiss law.

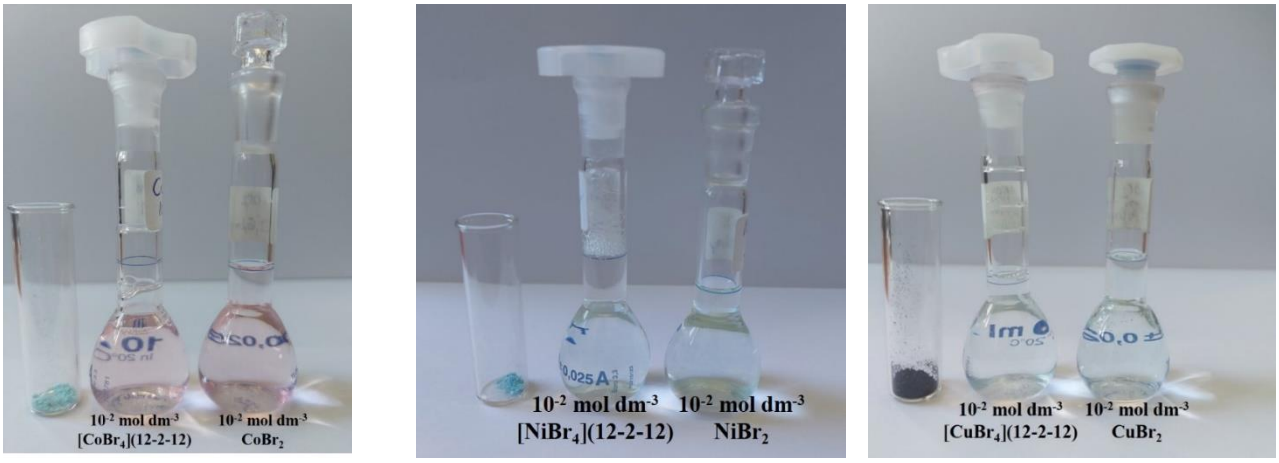

Fig. S9. Photos of colored dimeric metallosurfactants in powder form and in a 10^{-2} mol dm^{-3} solution, along with images of a 10^{-2} mol dm^{-3} solution of the corresponding metallic bromide used in the synthesis.

Table S9. Variation of average hydrodynamic diameter (d_h) of micelles with the concentration (c) for (12-2-12)[MBr_4] series of surfactants and metal-free precursor 12-2-12 at 25 ^oC. The results were obtained as a value at peak maximum of size volume distributions.

**c / mmol dm^{-3}**	**12-2-12**	**(12-2-12)[$CoBr_4$]**		**(12-2-12)[$NiBr_4$]**		**(12-2-12)[$CuBr_4$]**		**(12-2-12)[$ZnBr_4$]**	
	Peak I	Peak I	Peak II	Peak I	Peak II	Peak I	Peak II	Peak I	Peak II
	d_h / nm	d_h / nm	d_h / nm	d_h / nm	d_h / nm	d_h / nm	d_h / nm	d_h / nm	d_h / nm
**1.0**	4.5 ± 1.0	4.5 ± 0.4		4.2 ± 0.2		4.1 ± 0.4		5.6 ± 0.2	
**2.0**	3.5 ± 0.1	6.2 ± 0.2		5.0 ± 0.2		11.1 ± 0.4		4.9 ± 0.2	
**3.0**	2.8 ± 0.1	10.2 ± 0.4		12.9 ± 0.3		13.6 ± 1.5		10.3 ± 0.6	
**4.0**	2.8 ± 1.7	18.2 ± 3.7		16.8 ± 2.7		8.8 ± 3.9 (vol% 81.3 ± 13.1)	23.8 ± 2.6 (vol% 54.7 ± 13.1)	16.3 ± 1.5	
**5.0**	6.2 ± 2.3	6.0 ± 1.9 (vol% 84.6 ± 12.9)	21.1± 1.1 (vol% 23.1 ± 6.1)	6.1± 0.9 (vol% 77.4 ± 1.2)	22.2 ± 2.4 (vol% 61.2 ± 42.4)	5.8 ± 2.6 (vol% 83.7 ± 12.9)	21.5 ± 2.3 (vol% 19.5 ± 11.4)	5.8 ± 2.5 (vol% 84.8 ± 10.3)	18.7 ± 4.1 (vol% 52.5 ± 43.6)

References for SI:

(1) Sheldrick, G. M. *SHELXT* – Integrated Space-Group and Crystal-Structure Determination. *Acta Crystallogr A Found Adv* **2015**, *71* (1), 3–8. https://doi.org/10.1107/S2053273314026370.
(2) Sheldrick, G. M. Crystal Structure Refinement with *SHELXL*. *Acta Crystallogr C Struct Chem* **2015**, *71* (1), 3–8. https://doi.org/10.1107/S2053229614024218.
(3) Dolomanov, O. V.; Bourhis, L. J.; Gildea, R. J.; Howard, J. A. K.; Puschmann, H. *OLEX2* : A Complete Structure Solution, Refinement and Analysis Program. *J Appl Crystallogr* **2009**, *42* (2), 339–341. https://doi.org/10.1107/S0021889808042726.
(4) Spek, A. L. Single-Crystal Structure Validation with the Program *PLATON*. *J Appl Crystallogr* **2003**, *36* (1), 7–13. https://doi.org/10.1107/S0021889802022112.
(5) Macrae, C. F.; Sovago, I.; Cottrell, S. J.; Galek, P. T. A.; McCabe, P.; Pidcock, E.; Platings, M.; Shields, G. P.; Stevens, J. S.; Towler, M.; Wood, P. A. *Mercury 4.0* : From Visualization to Analysis, Design and Prediction. *J Appl Crystallogr* **2020**, *53* (1), 226–235. https://doi.org/10.1107/S1600576719014092.
(6) Mugiraneza, S.; Hallas, A. M. Tutorial: A Beginner's Guide to Interpreting Magnetic Susceptibility Data with the Curie-Weiss Law. *Commun Phys* **2022**, *5* (1), 95. https://doi.org/10.1038/s42005-022-00853-y.
(7) Yang, L.; Powell, D. R.; Houser, R. P. Structural Variation in Copper(I) Complexes with Pyridylmethylamide Ligands: Structural Analysis with a New Four-Coordinate Geometry Index, τ_4. *Dalton Trans.* **2007**, No. 9, 955–964. https://doi.org/10.1039/B617136B.
